# E3 ubiquitin ligase Herc3 deficiency leads to accumulation of subretinal microglia and retinal neurodegeneration

**DOI:** 10.1038/s41598-024-53731-8

**Published:** 2024-02-06

**Authors:** Yeshumenesh Zegeye, Bogale Aredo, Seher Yuksel, Dogan Can Kirman, Ashwani Kumar, Bo Chen, Emily Turpin, Sangita Shresta, Yu-Guang He, Laurent Gautron, Miao Tang, Xiaohong Li, Sophia M. DiCesare, John D. Hulleman, Chao Xing, Sara Ludwig, Eva Marie Y. Moresco, Bruce A. Beutler, Rafael L. Ufret-Vincenty

**Affiliations:** 1 Department of Ophthalmology, UT Southwestern Medical Center, Dallas, TX USA; 2 McDermott Center for Human Growth and Development, UT Southwestern Medical Center, Dallas, TX USA; 3 Center for Hypothalamic Research and Department of Internal Medicine, University of Texas Southwestern Medical Center, Dallas, TX 75390 USA; 4 Center for the Genetics of Host Defense, University of Texas Southwestern Medical Center, Dallas, TX USA; 5 Department of Bioinformatics, UT Southwestern Medical Center, Dallas, TX USA; 6 Present Address: Department of Ophthalmology, Tongji Hospital, Tongji Medical College, Huazhong University of Science and Technology, Wuhan, Hubei China

**Keywords:** Herc3, Forward genetics, ENU-mutagenesis, CRISPR, Retinal neurodegeneration, Fundus spots, Screening, Phenotype-genotype association, Microglia, Retinal diseases, Transcriptomics, Animal disease models

## Abstract

Activated microglia have been implicated in the pathogenesis of age-related macular degeneration (AMD), diabetic retinopathy, and other neurodegenerative and neuroinflammatory disorders, but our understanding of the mechanisms behind their activation is in infant stages. With the goal of identifying novel genes associated with microglial activation in the retina, we applied a semiquantitative fundus spot scoring scale to an unbiased, state-of-the-science mouse forward genetics pipeline. A mutation in the gene encoding the E3 ubiquitin ligase Herc3 led to prominent accumulation of fundus spots. CRISPR mutagenesis was used to generate *Herc3*^*-/-*^ mice, which developed prominent accumulation of fundus spots and corresponding activated Iba1 + /CD16 + subretinal microglia, retinal thinning on OCT and histology, and functional deficits by Optomotory and electrophysiology. Bulk RNA sequencing identified activation of inflammatory pathways and differentially expressed genes involved in the modulation of microglial activation. Thus, despite the known expression of multiple E3 ubiquitin ligases in the retina, we identified a non-redundant role for Herc3 in retinal homeostasis. Our findings are significant given that a dysregulated ubiquitin–proteasome system (UPS) is important in prevalent retinal diseases, in which activated microglia appear to play a role. This association between Herc3 deficiency, retinal microglial activation and retinal degeneration merits further study.

## Introduction

While there is extensive literature documenting the presence of activated microglia in the retina of patients with age-related macular degeneration (AMD), retinal dystrophies and diabetic retinopathy, our understanding of their role in these disease processes is still in infant stages^1^. It is clear that activated retinal microglia can have both pathogenic effects (e.g. secretion of proinflammatory molecules) and homeostatic effects (e.g. clearance of debris and dying cells)^2–17^. However, we still do not understand what controls the balance between these two potential behaviors. Because of the complexity of this biological system, we chose to exploit the unbiased nature of a forward genetics approach to search for novel genes modulating retinal immune cell activation. We identified an E3 ubiquitin ligase as a gene of interest.

As the name implies, ubiquitin is a protein that is expressed in all eukaryotic cell types and tissues. The ubiquitin proteasome system (UPS) is a complex degradation system that regulates protein function, localization, as well as stability^18^. It is one of the major systems in charge of removing damaged proteins and organelles, including dysfunctional mitochondria^19^. The UPS has been shown to be involved in photoreceptor cell survival and homeostasis^20–23^, and many prevalent retinal diseases appear to involve the UPS. Deficiencies of several UPS pathway components have been associated with defects in retinal development or homeostasis leading to forms of retinitis pigmentosa^18^. Moreover, in AMD, a very prevalent blinding retinal degeneration, disrupted lysosomal clearance and increased accumulation of waste products have been shown to be important pathophysiologic factors^24–26^. Finally, in diabetic retinopathy, hyperglycemia, oxidative stress, hypoxia and inflammation lead to dysregulation of the UPS in the retina^27^. Thus, modulation of specific components of the UPS in different directions may have therapeutic applications for some of the most common and vision-stealing retinal diseases^28^.

Three types of enzymes play a role in ubiquitination: ubiquitin activating enzymes (E1s), ubiquitin conjugating enzymes (E2s), and ubiquitin ligases (E3s)^20,29,30^. There are two known E1 ligases and about 30–40 E2 ligases^18^. In contrast, there are close to one thousand E3 ubiquitin-ligase enzymes^31,32^, which direct the process by targeting ubiquitin to specific proteins. E3 ubiquitin ligases are categorized into four families: the RING finger, SCF, APC and HECT (homologous to the E6-AP carboxyl terminus) families^33,34^. Of interest, while defects in E3 ligases may lead to retinal degeneration via multiple mechanisms, overexpression of E3 ligases may also be of therapeutic value in some retinal diseases like retinal dystrophies^35^.

In this work, we used a state-of-the-science forward genetics pipeline in combination with a fundus photography scoring scale to identify genes modulating the accumulation of yellow fundus spots, which we and others have shown often correlate with subretinal microglia^3,4,36–39^. We identified a mutation in *Herc3* that led to a prominent accumulation of yellow fundus spots. The human *HERC3* gene codes for a 117-kDa E3 ubiquitin ligase containing a HECT domain. It is expressed in most cell types and shows particularly high mRNA levels in the brain^40,41^. In mice, *Herc3* is also prominently expressed in the brain, but there is no published work showing HERC3 activity in the retina. Next, we generated *Herc3*^*-/-*^ mice using CRISPR-mediated gene editing and corroborated that they developed a prominent accumulation of activated subretinal microglia, retinal degeneration, and a decrease in retinal function. Finally, retinal transcriptomic analysis revealed activation of immune system-related pathways in *Herc3*^*-/-*^ mice. This study is the first one to not only demonstrate that HERC3 is expressed in the retina, but that despite the presence of many E3 ligases in retinal cells, HERC3 has a non-redundant role in retinal homeostasis. A better understanding of the role of HERC3 in the retina may be helpful in developing therapies to address retinal disorders associated with UPS dysfunction/dysregulation. This new model may also help us further understand the role of subretinal microglia in retinal physiology and disease.

## Results

### Screening of a forward genetics pipeline using a fundus spot semiquantitative scale identifies a gene coding for an E3 ubiquitin ligase

**Figure 1:**
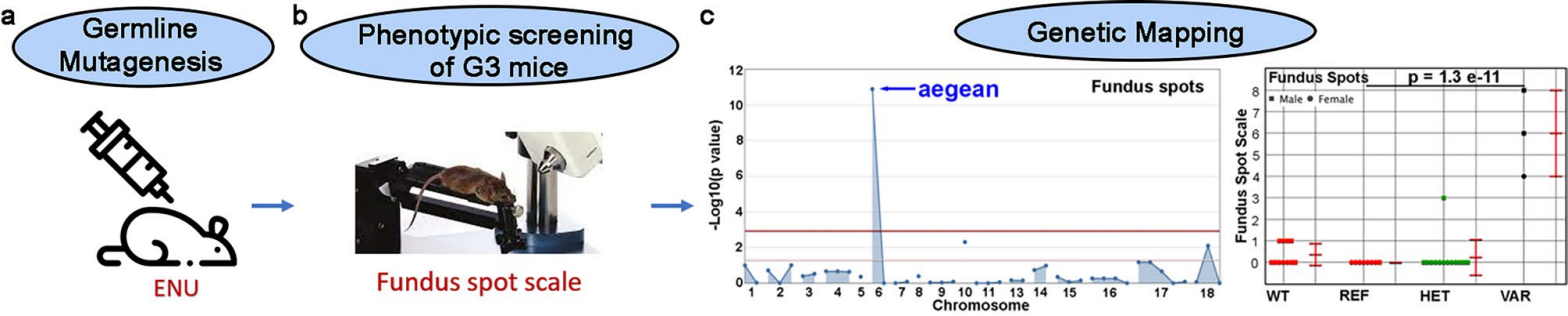
Forward genetics screening identified an allele (aegean) with a novel association to retinal homeostasis. (**a**) Random germline mutations were induced in C57BL/6 J mice using N-ethyl-N-nitrosourea (ENU). For each G1 founder mice, whole exome sequencing was performed before breeding it to generate G3 mice. The zygosity of each of the known mutations in its pedigree was determined for each of the G3 mice. (**b**) All G3 mice within the pedigree were then screened using retinal imaging. (**c**) In one of the pedigrees studied, genetic mapping using specialized software (Linkage Analyzer) identified a strong association (p = 1.3 × 10^–11^) between the aegean allele and fundus spot accumulation. This was the only mutation in this pedigree showing an association to fundus spots, as shown by the single peak in the Manhattan plot (left panel). All three mice homozygous for the aegean allele (VAR) in this pedigree showed a marked increase in fundus spots (right panel). Heterozygous mice (HET) did not show an abnormality. WT, C57BL/6 J; REF, homozygous for the C57BL/6 J reference allele. Data are presented as mean ± SD (WT, n = 11, HET, n = 13, REF, n = 8, VAR, n = 3). Statistical analysis was done using two-tailed ANOVA. The mice were 6–8 months old at the time of screening.

Forward genetics approaches have been helpful in identifying genes involved in retinal degeneration^42–46^. Our robust and validated forward genetic pipeline (Fig. 1)^47,48^ is based on ENU mutagenesis of C57BL/6 J mice. Founder (G0) mice undergo whole exome sequencing, which then allows for genotyping of all third generation (G3) mice at all mutant loci prior to screening. This is an advantage compared to other forward genetics protocols and it allows for immediate determination of causative mutations after screening protocols^48^. Aiming to identify novel genes with an impact on retinal immune cell activation, we developed^3^ a semiquantitative fundus spot scoring scale and applied it to this forward genetics pipeline. So far, we have scored close to 11,800 fundus photographs obtained from 5906 4–6-month old G3 mice carrying heterozygous and homozygous ENU-induced mutations^47,48^. This corresponds to a 4.8% saturation of the genome (considering genes with probable null or damaging mutations, and at least 2 mice in the homozygous state). Automated meiotic mapping^48^ using the Linkage Analyzer software helped us find links between the phenotype of mice showing fundus spot accumulation and specific genetic mutations. This was followed by the application of a machine learning algorithm (Candidate Explorer software) that predicts the likelihood of being able to reproduce the phenotype if the identified gene is independently targeted^49^. We identified a nonsense mutation (glutamine 137 to stop) in the *Herc3* gene, which codes for an E3 ubiquitin ligase. This allele, which we named *aegean*, leads to early termination of the HERC3 protein at amino acid 137 (normally 913 aa), with a predicted null effect. Of note, the mouse Herc3 protein has 95.4% similarity to human HERC3 and is conserved at the site of this mutation. While there were only 3 mice homozygous for the mutation in the affected pedigree, each of them showed a significant increase in fundus spots, leading to a very strong association (Fig. 1c, p = 1.3 × 10^–11^). The combination of a single peak in the Manhattan plot (Fig. 1c), plus the fact that all mice homozygous for the *aegean* allele exhibited the phenotype (Fig. 1c) provided reassurance that this *Herc3* mutation was the only mutation responsible for the observed phenotype. Mice heterozygous for the mutation did not show increased accumulation of fundus spots (Fig. 1c), indicating an autosomal recessive trait.

### *Herc3*^*-/-*^ mouse lines develop progressive fundus spot accumulation

To confirm that the observed phenotype was indeed caused by the *Herc3* mutation, we used CRISPR/Cas9 mutagenesis to generate two *Herc3*^*-/-*^ mouse lines with almost identical mutations. Both have a 1 bp insertion at the same location of exon 14 (either an A or a T) creating a frameshift that is immediately followed by a premature stop codon (see Methods and Supplementary Fig. S1; the normal length of HERC3 is 913 aa). Moreover, their transcripts are predicted to be subject to nonsense mediated decay. We confirmed this by showing a strong decrease in *Herc3* transcript in the *Herc3*^*-/-*^ mice by qPCR (Supplementary Fig. S2a). It was further confirmed in a bulk RNAseq retinal transcription analysis (Supplementary Fig. S2b). Both *Herc3*^*-/-*^ mouse lines were healthy, fertile, and displayed no gross abnormalities. We did not find any significant differences in weight between *Herc3*^*-/-*^ and *Herc3*^+*/*+^ mice (Supplementary Fig. S3).

**Figure 2:**
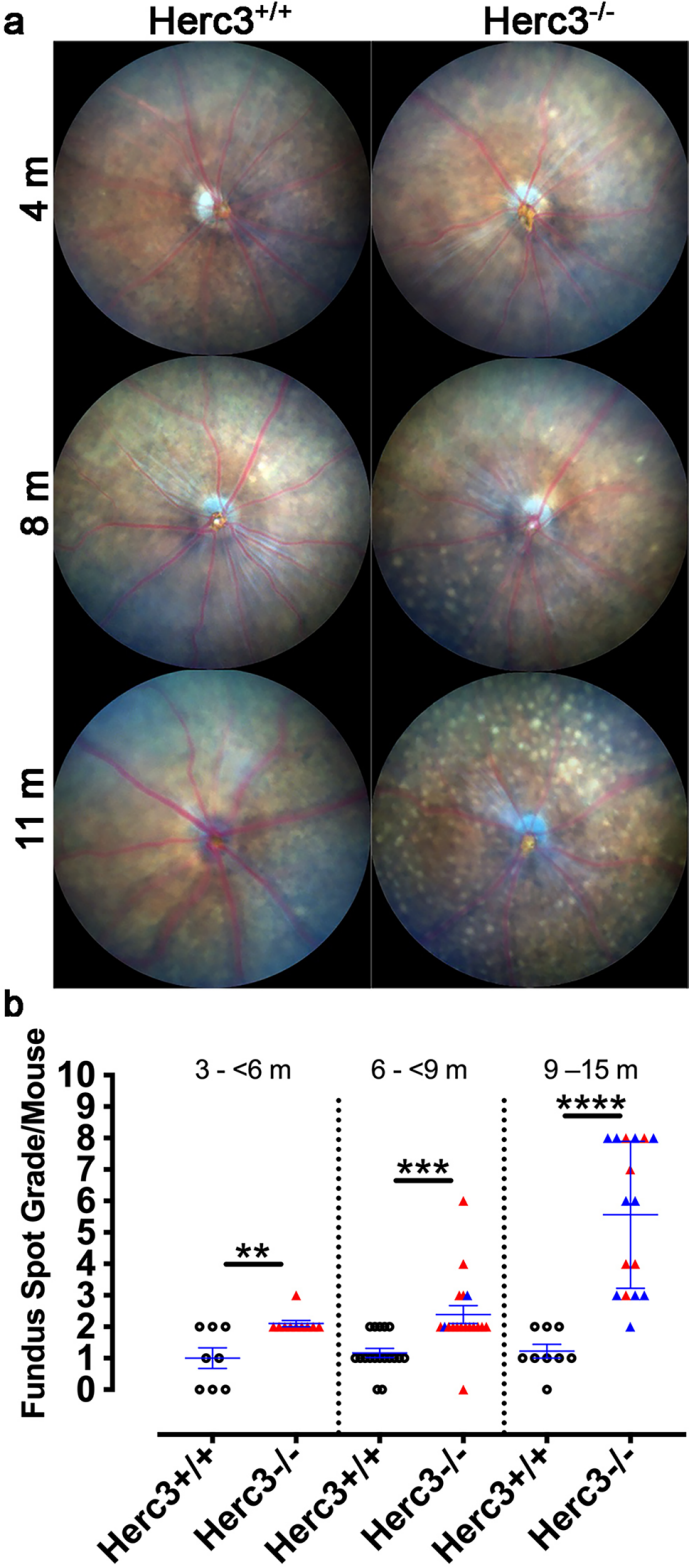
Prominent buildup of fundus spots in mice deficient in *Herc3*. (**a**) Fundus photographs of *Herc3*^+*/*+^ and *Herc3*^*-/-*^ mice were obtained at 4 m, 8 m, and 11 m of age. (**b**) Semi-quantitative fundus spot grading shows a statistically significant increase in fundus spots in mutant mice compared to control mice at three different ages (3- < 6 m; 6- < 9 m; 9–15 m; *Herc3*^+*/*+^, n = 8, 16, 10 and *Herc3*^*-/-*^, n = 10, 18, 14 respectively). Data are shown as Means ± SEM. Two-tailed student’s t-test: **p < 0.01, ***p < 0.001, ****p < 0.0001. Colored symbols represent *Herc3*^*-/-*^ mice from either the Herc3(T) line (Red colour filled triangle) or the Herc3(A) line (Blue colour filled triangle).

We proceeded to take fundus images of *Herc3*^*-/-*^ vs. *Herc3*^+*/*+^ mice. Qualitative analysis (Fig. 2a) shows a very strong pattern of progressive fundus spot accumulation in the *Herc3*^*-/-*^ mice as they age. Of note, no difference in fundus spot accumulation between the two *Herc3*^*-/-*^ lines was found (Supplementary Fig. S4a). However, comparison of each one of the knockout lines to wild type mice did in fact show a statistically significant increase in fundus spots. Thus, *Herc3*^*-/-*^ mice of both knock out lines [Herc3(A) and Herc3(T)] were combined for analyses.

Using the same fundus spot scale that we applied to the forward genetics screen, two investigators masked to genotype scored the *Herc3*^*-/-*^ and *Herc3*^+*/*+^ fundus images. Statistically significant differences between *Herc3*^*-/-*^ and *Herc3*^+*/*+^ mice (Fig. 2b) were observed at 3–6 m of age (p = 0.0028), 6–9 m of age (p = 0.00049) and 9–14 m of age (p = 2.2 × 10^–5^). Of note, analyzing the data using only the Herc3(T) line (red triangles) led to statistically significant differences (p < 0.01 for all time points). However, we included mice from the Herc3(A) line (blue triangles) just to show that their phenotype is similar to the Herc3(T) line. The magnitude of the difference increased as the mice aged (Fig. 2b). A linear regression analysis corroborated this age-dependent increase in spots in *Herc3*^*-/-*^ mice (R^2^ = 0.59; F = 47.4, p = 7.3 × 10^–8^; see Supplementary Fig. S4b).

### Deficiency of Herc3 leads to accumulation of activated subretinal microglia

Previous studies have linked the clinical appearance of yellow/white fundus spots on retinal photos to the accumulation of sub-retinal Iba1 + cells (a pan-marker of microglia/macrophages)^3,4,36–39^. We performed immunohistochemistry of both retinal and RPE-choroid-sclera flat mounts (referred to below as “RPE flat mounts”) and demonstrated the presence of large numbers of Iba1 + /CD16 + cells in the subretinal space of *Herc3*^*-/-*^ but not *Herc3*^+*/*+^ mice (Supplementary Fig. S5). In separate eyes we stained for F4/80 (another pan-marker of microglia/macrophages) and TMEM119 (specific for microglia). We found many F4/80 + , TMEM119 + cells in the outer retina and subretinal space of *Herc3*^*-/-*^ but not *Herc3*^+*/*+^ mice. Interestingly, all subretinal cells positive for F4/80 were also positive for TMEM119 (Fig. S5 bottom right panel). These cells were also negative for the infiltrating macrophage marker CCR2 (Fig. S6).

A one-to-one correlation of white/yellow spots in fundus photos versus Iba1 + microglia in flat mounts is not possible for several reasons, including the fact that a fundus photo is a 2-dimensional representation of a spherical surface, while the flat mount utilizes radial cuts to flatten the spherical retina in a nonuniform manner. Still, in our mice there seemed to be a general spatial correlation of fundus spots and Iba1 + subretinal cells (Supplementary Fig. S7).

**Figure 3:**
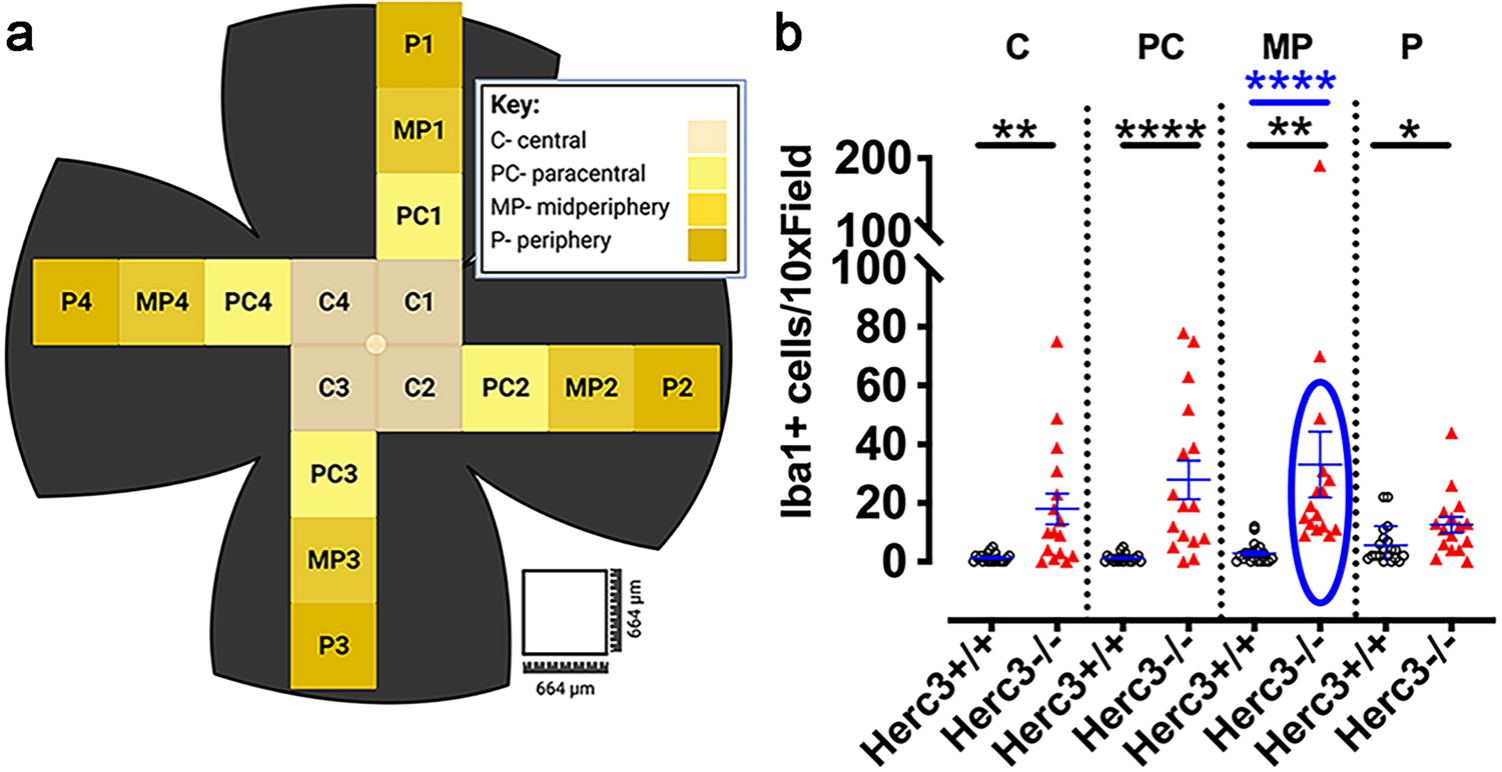
Quantification of subretinal microglia in *Herc3*^*-/-*^. Eyes were collected for RPE/choroid/sclera flat mounts (n = 5 *Herc3*^+*/*+^ and 4 *Herc3*^*-/-*^ eyes) and stained with an anti-Iba1 antibody mice. (**a**) Iba1 + cells were counted at 4 different antero-posterior regions of the flat mounts (labeled as central, paracentral, mid-peripheral and peripheral regions – diagram generated using BioRender.com). (**b**) *Herc3*^*-/-*^ demonstrated a statistically significant increase in Iba1^+^ cells in each of the quantified regions. The difference was significant whether two outliers were included or excluded (the blue oval and asterisks show analysis with exclusion – the interquartile range technique was used to determine outliers, as described in “Methods”). Data are shown as means ± SEM. Two-tailed student’s *t* test: *p < 0.05, **p < 0.01, ****p < 0.0001. Scale bar = 250 µm.

Quantitative analysis of the subretinal microglia was then performed. Iba1 + cells were counted at central, paracentral, midperipheral, and peripheral regions (Fig. 3a). We found a statistically significant increase in the number of Iba1 + cells in *Herc3*^*-/-*^ mice compared to *Herc3*^+*/*+^ (Fig. 3b) for the central (p = 0.0011), paracentral (p = 6.5 × 10^–5^), mid-peripheral (p = 0.0047) and peripheral (p = 0.023) regions.

### Optical coherence tomography (OCT) and histological analysis demonstrate outer retinal thinning in *Herc3*^*-/-*^ mice

**Figure 4:**
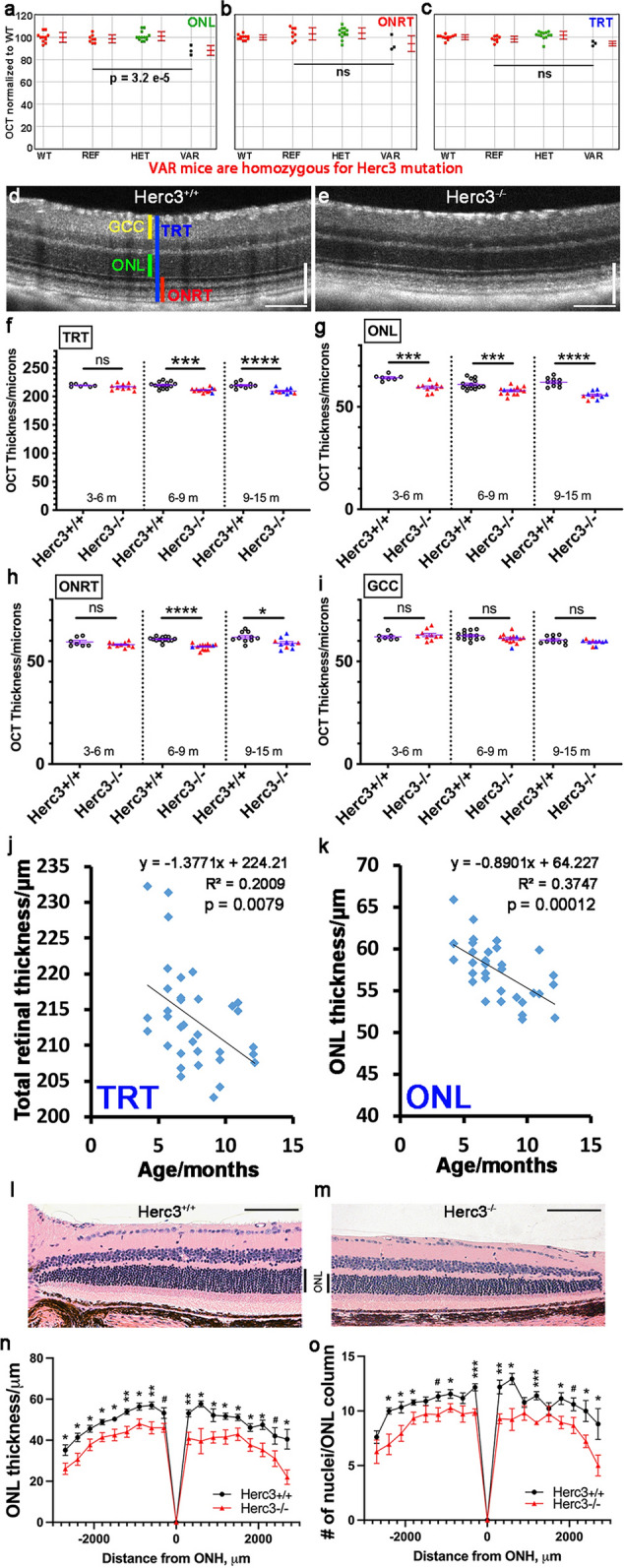
Progressive thinning of the outer retina is seen in *Herc3* deficient mice. Despite having only 3 mice homozygous for the aegean allele in the pedigree, the ENU-mutagenesis screen (**a**–**c**) revealed a statistically significant decrease in ONL (**a**). A representative OCT image of a 11.5 mo CRISPR-generated *Herc3*^*-/-*^ mouse (**e**) shows a thinner outer retina and qualitatively disrupted outer retinal layers compared to control (**d**). OCT images were analyzed using four parameters: Total retinal thickness (TRT, blue vertical line), Ganglion cell complex (GCC, yellow line), Outermost neural retina thickness (ONRT, red line), and outer nuclear layer (ONL, white line) (**d**). Quantitative analyses (**f**–**i**) show that the thinning of the ONL becomes statistically significant by 3–5 m of age (**g**), while that of the TRT and ONRT parameters becomes significant at 6–7 m of age (**f**,**h**). Meanwhile, no statistically significant changes are observed for the GCC parameter (**i**). For *Herc3*^+*/*+^, n = 7, 14 and 10 mice for the 3- < 6 m; 6- < 9 m; 9–15 m groups respectively, while for *Herc3*^*-/-*^, n = 10, 14 and 10 mice for the same age groups. In panels (**f**–**h**), colored symbols represent *Herc3*^*-/-*^ mice from either the Herc3(T) line (Red colour filled triangle) or the Herc3(A) line (Blue colour filled triangle). Linear regression analysis shows a statistically significant downward trend in TRT ((**j**); R^2^ = 0.2, F = 8.1, p = 0.0079) and ONL ((**k**); R^2^ = 0.374, F = 19.2, p = 0.00012). Each symbol represents a mouse. ONL thinning was also confirmed on H&E-stained retina sections (**l**-**o**). Representative Sects. (20X magnification) of a 10-m-old control (**l**) and an age-matched *Herc3*^*-/-*^ mouse (**m**) show thinning of the ONL in the mutant mouse. The thickness of the ONL (**n**) and the number of cells (nuclei) in the ONL (**o**) were measured on H&E sections at 300 µm intervals from the Optic Nerve Head (ONH). *Herc3*^*-/-*^ mice (n = 6) had a statistically significant thinner outer nuclear layer, based on both parameters, compared to control (n = 6). Two-tailed student’s t-test: *ns* not statistically significant, *P < 0.05, **P < 0.01, ***P < 0.001, ****P < 0.0001, and ^#^P < 0.1.

We had noticed a significant thinning of the ONL (Fig. 4a–c) in the original ENU-generated pedigree containing the *aegean* allele. To better evaluate the impact of Herc3 deficiency on the anatomy of the retina, we used a Phoenix MICRON OCT2 system, to evaluate our CRISPR-generated *Herc3*^*-/-*^ and *Herc3*^+*/*+^ mice at various ages (from 3 to 15 months). An analysis was done to compare the two knock out lines [Herc3(A) and Herc3(T)] and no OCT differences were seen (Supplementary Fig. S8a,b). Thus, further analyses were done combining mice of both knock out lines.

From qualitative analysis of the OCT pictures, we observed both a difference in reflectivity of the outer layers and an obvious reduction in the outer retinal thickness of the *Herc3*^*-/-*^ eyes as compared to the *Herc3*^+*/*+^ (see Fig. 4e vs 4d). Quantitative analysis was then performed using the following parameters: total retinal thickness (TRT), outer nuclear layer (ONL), outermost neural retina thickness (ONRT) and ganglion cell complex (GCC). We found that in the *Herc3*^*-/-*^ mice all the metrics that included the outer retina, namely the ONL, TRT, and ONRT, significantly decreased compared to *Herc3*^+*/*+^ mice (see Fig. 4f–h). The difference became significant at 3–5 m for the ONL and at 6–7 m for the TRT and ONRT. Meanwhile, there was no difference in the inner retina (GCC) at any age (Fig. 4i). Of note, we initially analyzed the TRT and ONL data using only the Herc3(T) line and it resulted in statistically significant differences (p < 0.01 for all time points). However, in the final analysis we included mice from the Herc3(A) line (blue symbols) to show that they have a similar phenotype to that of the Herc3(T) line. The outer retinal thinning in the *Herc3*^*-/-*^ mice progressed with age. A trend analysis of the OCT images in *Herc3*^*-/-*^ mice by age confirmed a significant negative trend for both TRT and ONL as these mice aged (Fig. 4j,k). Histological analysis on H&E-stained retina sections confirmed the thinning of the ONL (Fig. 4l–o). In order to determine if we could find evidence of increased apoptosis in the *Herc3*^*-/-*^ we performed a TUNEL assay on retinal sections. We only found a very small number of TUNEL + cells in both *Herc3*^*-/-*^ and *Herc3*^+*/*+^ mice, with no difference between the groups (Supplementary Fig. S9a). We also looked at the density of cones on retinal sections using cone arrestin staining (Supplementary Fig. S9b-d) and did not find a difference between *Herc3*^*-/-*^ and *Herc3*^+*/*+^ mice. Finally, using electron microscopy to evaluate the RPE we could only find minimal abnormalities of unknown significance (EM, Supplementary Fig. S10).

### Potential impact of sex as a biological variable

Re-evaluation of our data suggested a bimodal distribution of fundus spots in the older age group (Fig. 2b) and that led us to re-analyze our data after segregating mice by sex (Supplementary Fig. S11). Even with the lower number of mice per group, for most comparisons, a statistically significant increase in fundus spots was seen in *Herc3*^*-/-*^ compared to *Herc3*^+*/*+^ mice independent of sex (Supplementary Fig. S11a). However, despite the fact that our analysis was post-hoc and our experiments were not powered to look at the effect of sex, we did observe an increase in fundus spots in female *Herc3*^*-/-*^ mice compared to male *Herc3*^*-/-*^ mice (Supplementary Fig. S11b). The difference was statistically significant in 6–9 m old mice (p = 0.03) and showed a strong trend in 9–15 m old mice (p = 0.06). No difference was seen in wild type mice by sex. Given these findings we also decided to analyze the effect of sex on the ONL thickness data. As we expected, we found that *Herc3*^*-/-*^ mice had significant thinning of the ONL compared to *Herc3*^+*/*+^ independent of sex (Supplementary Fig. S11c). However, to our surprise, we also found thinning of the ONL in female *Herc3*^*-/-*^ mice compared to male *Herc3*^*-/-*^. The difference was statistically significant for the 3–6-month-old and for the 9–15-month-old age groups, and there was a trend (p = 0.09) in the 6–9-month-old age group (Supplementary Fig. S11d). No sex-related differences were seen in wild type mice.

### *Herc3*^*-/-*^ mice have decreased visual function as shown on optomotor responses and electrophysiology testing

**Figure 5:**
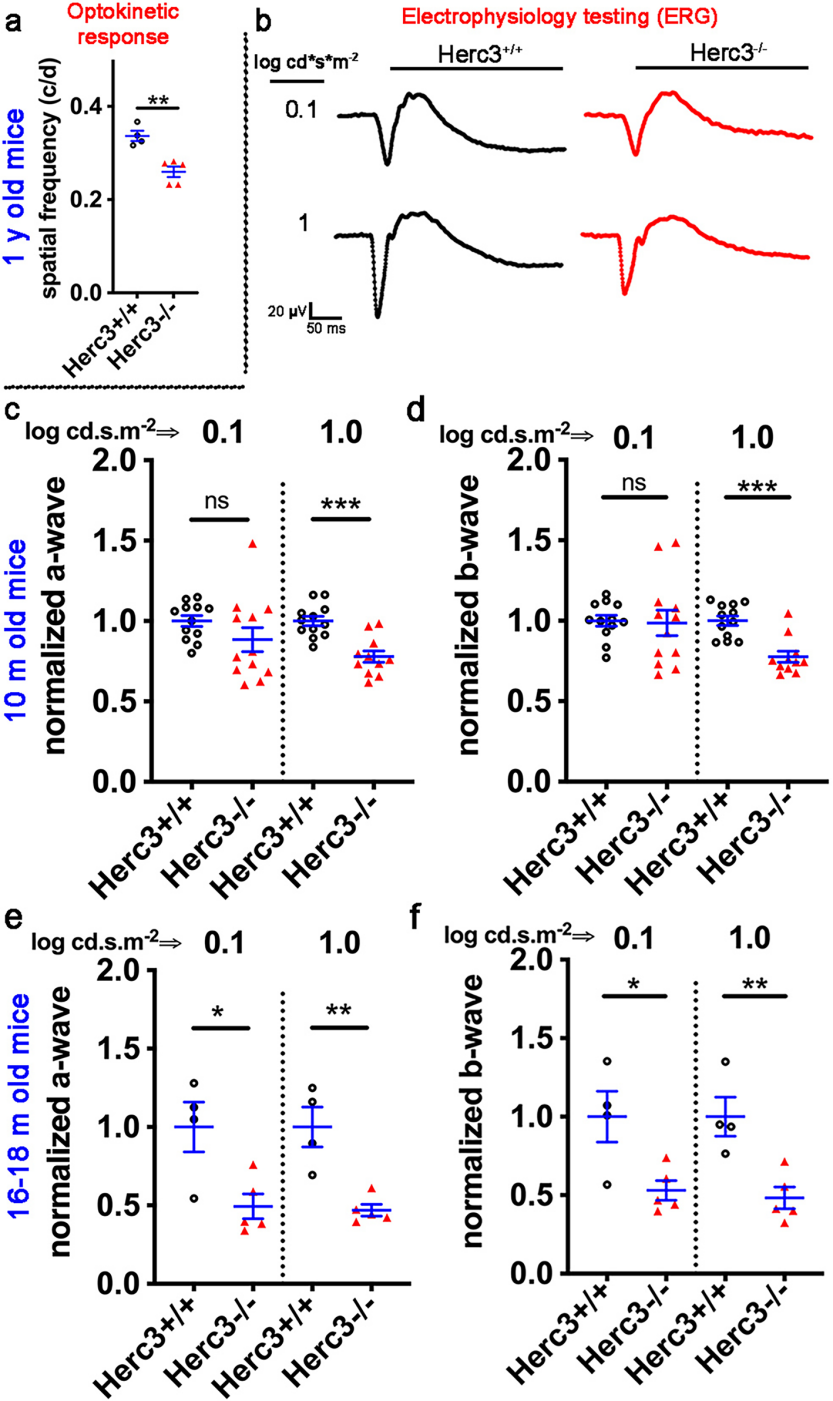
Optokinetic testing and electroretinography analysis show functional deficit in *Herc3*^*-/-*^ mice. (**a**) Spatial frequency threshold was determined on *Herc3*^*-/-*^ (n = 5) and *Herc3*^+*/*+^ (n = 4) mice using OptoMotry system and showed a significant decrease of spatial vision in *Herc3*^*-/-*^ mice (p = 0.002) compared to *Herc3*^+*/*+^ mice. (**b**) Scotopic Ganzfeld ERG a-wave and b-wave were measured in response to low (0.1 log cd.s.m^–2^) and high (1 log cd.s.m^–2^) flash intensities in 10-month-old mice. Representative ERG traces from *Herc3*^*-/-*^ and control mice are shown. Analysis of the ERG data showed a statistically significant reduction in both a-wave (**c**) and b-wave (**d**) signals in response to high flash intensity in *Herc3*^*-/-*^ (n = 12 mice) compared to control mice (n = 12 mice). Analyses were also done on 16–18 month-old mice (*Herc3*^+*/*+^, n = 4 and *Herc3*^*-/-*^, n = 5). Statistically significant decreases in the scotopic a-wave (**e**) and b-wave (**f**) were seen in these aged mice. Two-tailed student’s *t* test: *ns* no significant difference, ^#^p < 0.1, *p < 0.05, **p < 0.01, ***p < 0.001.

To investigate visual function, a masked investigator tested 1-year-old *Herc3*^+*/*+^ (n = 4) and *Herc3*^*-/-*^ mice (n = 5) using the OptoMotry system (Cerebral Mechanics, Inc., Lethbridge, AB, Canada). The spatial frequency threshold, a measure of visual behavior, showed a highly significant decrease in *Herc3*^*-/-*^ mice (Fig. 5a, p = 0.002) compared to *Herc3*^+*/*+^ mice. We then obtained full-field scotopic Ganzfeld ERG in 10-month-old *Herc3*^*-/-*^ and *Herc3*^+*/*+^ mice after 12–16 h of dark adaptation. Representative traces from both *Herc3*^*-/-*^ and control mice after low (0.1 log cd.s.m^-2^) and moderate (1 log cd.s.m^-2^) flash stimuli are shown (Fig. 5b). Quantification showed statistically significant reductions in both the a-wave (Fig. 5c, p = 9.0 × 10^–5^) and b-wave (Fig. 5d, p = 7.4 × 10^–5^) of *Herc3*^*-/-*^ mice in response to the moderate light intensity stimulus. These differences increased in magnitude with aging (16–18 m old mice, Fig. 5e and f). Interestingly, minimal to no differences were seen in c wave, oscillatory potentials (OP) and photopic a and b waves (Supplementary Fig. S12).

### Herc3 is ubiquitously expressed in the retina: single-cell RNA sequencing and RNAscope

**Figure 6:**
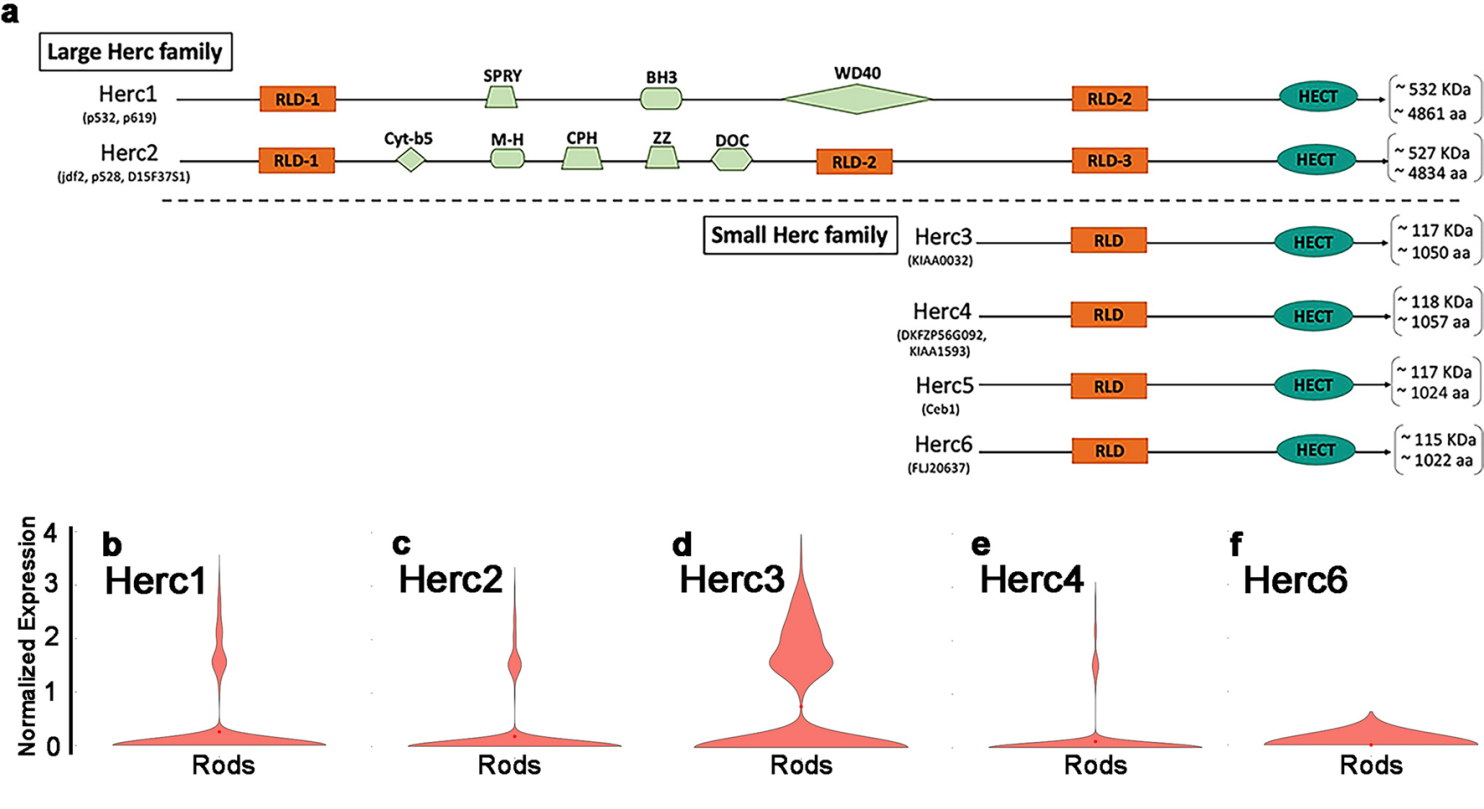
Single-cell RNA-seq data in C57BL/6 J mice show that *Herc3* is the only *Herc* family member with significant expression in rod photoreceptors. (**a**) Diagram of the *Herc* family proteins. The *Herc* family of proteins is grouped by size: large members include *Herc1* and *Herc2*, while the phylogenetically distinct small members include *Herc3-Herc6*. *Herc5* is not expressed in mice. The large *Herc* family members have a single HECT domain, more than one RLD domain, and additional units. The small *Herc* family members contain a single HECT domain and only one RLD domain. (**b**-**f**) *Herc3* is the only family member showing significant expression in rod photoreceptors.

Herc3 is part of a family of proteins (Fig. 6a) which include two large members (Herc1 and Herc2) and four small members (Herc3-Herc6)^40,50^. To determine the expression pattern of *Herc3* (and other Herc family members) in the retina, we performed single cell RNA sequencing in young C57BL/6 J mice (4 m old). The mean expression of each Herc family member for each cell type, and the percentage of each cell type expressing them are shown in Supplementary Table S2. *Herc3* was the only *Herc* molecule significantly expressed in rod photoreceptors (Fig. 6b–f and Supplementary Table S2). In fact, Herc3 is expressed, at moderate levels, in several retinal cell types studied including rod photoreceptors, amacrine cells, bipolar cells, horizontal cells and retinal ganglion cells (Supplementary Table S2 and Fig. S13a–e). Both of the large Herc family members (Herc1 and Herc2) are ubiquitously expressed at levels similar to Herc3. Meanwhile, none of the small Herc family members apart from Herc3 are significantly expressed. UMAP analysis showing the expression of Herc3 in the 23 clusters we identified helps visualize the ubiquitous expression of Herc3, with strongest levels in rod photoreceptors, but also several clusters of bipolar and amacrine cells (Fig. S13f). Interestingly, in situ hybridization using RNAscope in wild type mice also confirmed the scRNA sequencing data, showing expression of Herc3 in multiple retinal layers (Fig. S13g). The staining was particularly prevalent and strong in the ONL (photoreceptors), but there was also significant staining in the INL (where bipolar cells and amacrine cells, among other cell types are found) and even in the RGC layer (Fig. S13g). A probe for Staphylococcus aureus Cas9 was used as a negative control. (Fig. S13h).

An analysis using the scRNA sequencing data tool from the publicly available NEI database eyeIntegration (https://eyeintegration.nei.nih.gov)^51^ shows that during development there is some expression of Herc3 in multiple cell types, but it is highest in rods and cones (Supplementary Fig. S14). In adult mice, the expression increases in several cell types, including amacrine cells, bipolar cells, retinal ganglion cells, photoreceptors and RPE. It is highest in rods, cones and rod bipolar cells. Microglia, macrophages, Muller cells, horizontal cells and astrocytes show lower expression.

### Herc3 deficiency leads to activation of inflammatory pathways in the retina: bulk-RNA sequencing

**Figure 7:**
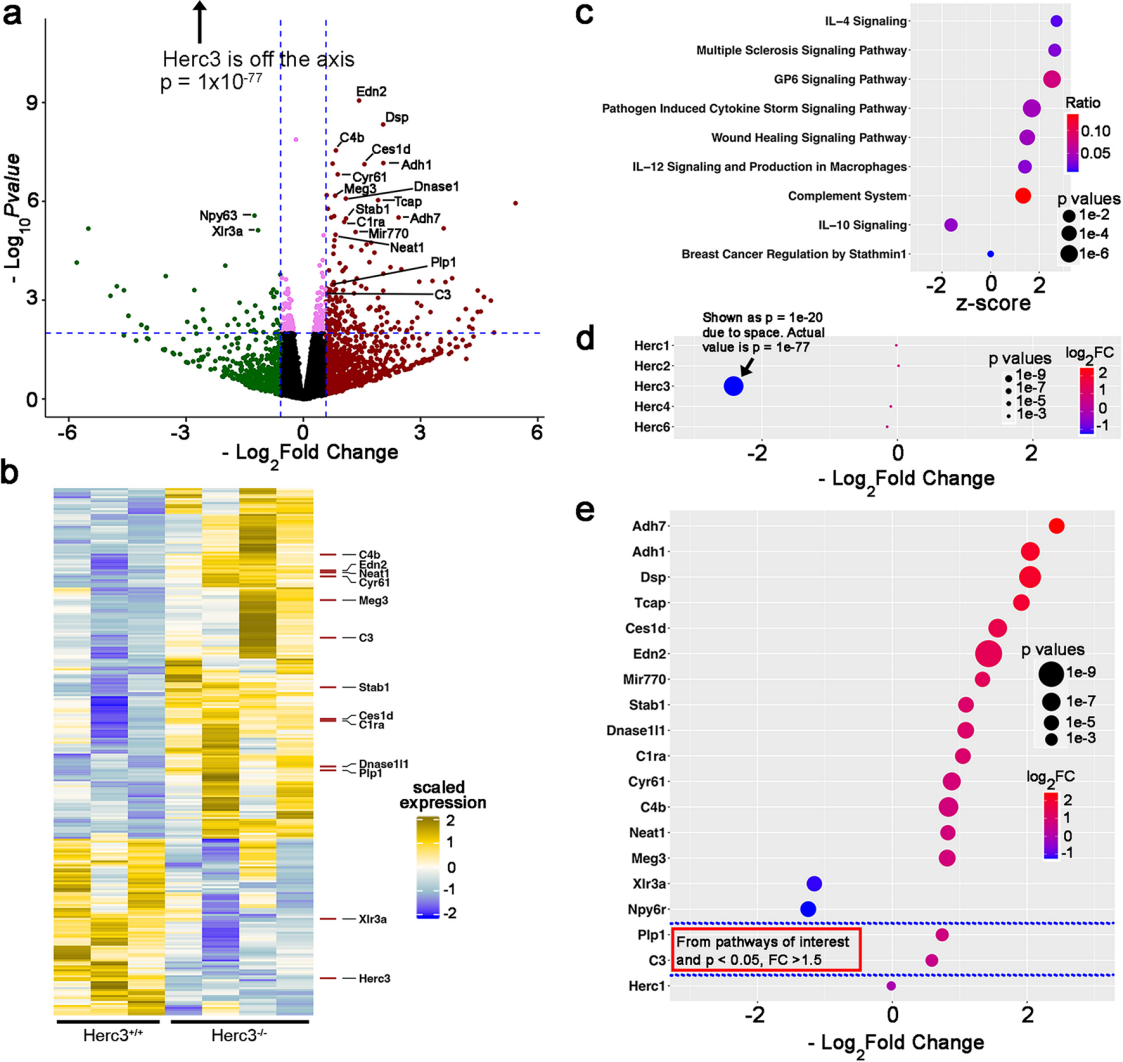
Bulk-RNAseq analysis shows activation of inflammatory pathways and differentially expressed genes related microglial activation in *Herc3*^*-/-*^ mice compared to controls. (**a**) A volcano plot shows *Herc3* is significantly down regulated in *Herc3*^*-/-*^ retina. Other genes were also differentially expressed. (**b**) Heat map shows different gene expression profile between *Herc3*^*-/-*^ and *Herc3*^+*/*+^ retina. (**c**) Results from the IPA show that pathways related to inflammation are activated. (**d**) Herc3^-/-^ is the only Herc family gene to be affected in Herc3^-/-^ retina. (**e**) Several genes related to the modulation of microglia cell activation are upregulated in Herc^-/-^ retinas compared to *Herc3*^+*/*+^. Samples consisted of both retinas of a mouse. Retina transcriptome data was obtained from 4 Herc3^-/-^ samples and 3 *Herc3*^+*/*+^ samples (7–8 month old).

To better understand the mechanisms behind the anatomic and functional abnormalities caused by the Herc3 deficiency, we performed a bulk RNA sequencing experiment using retina of 7–8 m old *Herc3*^*-/-*^ vs *Herc*^+*/*+^ mice. A volcano plot (Fig. 7a) revealed 192 genes that were upregulated (p < 0.01 and log_2_FC > 0.58, equivalent to a fold change of > 1.5), while 65 genes were downregulated (p < 0.01 and log_2_FC < -0.58) in *Herc3*^*-/-*^ mice.

A heat map was generated after filtering for genes with a p value < 0.01 and logCPM > 0, resulting in 266 genes (Fig. 7b). Our genes of interest are labeled. Pathway analysis was done with IPA using these 266 genes to generate a list of significantly changed pathways. We chose relevant pathways showing a p value < 0.05 and |Z score|> 1.3). Most of the resulting pathways (Fig. 7c) involved aspects of inflammation: cytokine pathways (“IL-4 signaling”, “IL-10 signaling” and “IL-12 signaling”), “Multiple Sclerosis Signaling Pathway”, “Pathogen Induced Cytokine Storm Signaling Pathway” and “Complement system”. Other pathways were the “GP6 Signaling Pathway” and the “Wound Healing Signaling Pathway”.

We started the gene-based analysis of our data by looking at the Herc family members and showing that Herc3 was the only differentially expressed family member in *Herc3*^*-/-*^ mice (Fig. 7d, the size of the circle for *Herc3* represents a p value of 1 × 10^–20^ instead of the actual p value of 1 × 10^–77^ due to space considerations). To search for genes of interest, we analyzed the bulk RNA seq data using two methods. First, we did an unbiased analysis filtering the results using as criteria an FDR < 0.01 and a fold change > 1.5. This resulted in 16 genes of interest (see top 16 genes in Fig. 7e). Our second analysis used IPA. From the list of molecules within the pathways of interest generated by IPA we selected genes that in our experiment had shown a differential expression with a p value < 0.05 and a fold change > 1.5. This analysis added the genes *C3* and *Plp1* to the prior list (between the blue lines in Fig. 7e). Herc1 is included as a negative control.

Interestingly, reviewing our identified differentially expressed genes we again observed strong evidence that Herc3 deficiency had an impact on inflammatory processes. Out of the top 20 differentially expressed genes in *Herc3*^*-/-*^ retinas (organized by p value), 7 have been associated to modulation of inflammation (see Supplementary Table S3). Several complement components were increased (e.g. C1ra, C3 and C4b), suggesting activation of the complement system. Some examples of differentially expressed genes that may be involved in modulating the immune system include *Neat1* (FC = 1.77, p value = 1 × 10^–5^), *Meg3* (FC = 1.76, p value = 6.7 × 10^–7^), *Edn2* (FC = 2.7, p = 9 × 10^–10^), Cyr61 (FC = 1.84, p value = 1.5 × 10^–7^), and *Npy6r* (FC = 0.42, p = 2.7 × 10^–6^).

## Discussion

We and others have shown that the accumulation of subretinal microglia can be seen as an accumulation of yellow fundus spots^3,4,36–39^. Thus, we used a semiquantitative fundus spot scale to screen for gene mutations leading to accumulation of fundus spots. We chose to pursue one of our potential “hits”, *Herc3*, because it showed a strong phenotype, it is novel, and it has an interesting E3 ubiquitin ligase function.

Due to the high metabolic activity and oxidative stress level in the retina-RPE, the ubiquitin–proteasome system (UPS) plays a very important role in maintaining homeostasis. It is essential in regulating the cell cycle, immune response, endoplasmic reticulum-associated degradation of misfolded or damaged proteins and elimination of damaged organelles. Multiple studies implicate UPS deficiencies or dysregulation in the pathogenesis of prevalent retinal diseases, including AMD, retinal dystrophies, and diabetic retinopathy^25,26,33,52,53^. Due to the high number of E3 ligases in the retina, one may expect significant redundancy. However, here we show for the first time that the E3 ligase Herc3 has an essential and irreplaceable role in retinal homeostasis.

Most importantly, analysis of the *Herc3*^*-/-*^ mouse lines yielded evidence of a progressive retinal neurodegeneration affecting mostly the outer retina, as documented by our analyses of several OCT parameters and histology. Moreover, this led to a statistically significant reduction in visual function as measured by optomotor responses (spatial single frequency thresholds) and ERG. It is likely that, while the fundus spots helped us identify Herc3 as an essential gene for retinal health, the retinal degeneration may be the most important and perhaps primary effect of the deficiency of Herc3. While it is possible that Herc3 deficiency could also be directly responsible for the microglial activation, similar activation of retinal microglia is seen in other models of retinal degeneration.

*Herc3*^*-/-*^ mice do show increased accumulation of subretinal Iba1 + /CD16 + cells, evidence that these subretinal cells are activated microglia/macrophages. A separate staining also showed accumulation of F4/80 + /TMEM119 + /CCR2- cells in RPE flat mounts of *Herc3*^*-/-*^ mice, indicative of subretinal microglia. While it seems that the majority of activated subretinal cells in our model are microglia, it is not possible to completely rule out some contribution of infiltrating macrophages. Further studies may be needed to determine whether microglial activation is contributing to or more likely trying to minimize the changes caused by the retinal degeneration.

Our bulk RNAseq data provides evidence of activation of inflammatory pathways in the retina of *Herc3*^*-/-*^ mice. However, it is possible that this is due to a chronic and low-grade reactive parainflammatory response trying to re-establish homeostasis^54^, as we did not see any evidence of overt inflammation clinically or histologically. Pathway analysis did reveal upregulation of IL-12 and IL-4 signaling, and downregulation of IL-10 signaling. IL-12 and IL-10 are both expressed by activated microglia/macrophages. They are reciprocally controlled and have opposing pro- and anti-inflammatory roles. Meanwhile, IL-4 is often expressed by neurons in response to the pro-inflammatory environment induced by IL-12^55^. Moreover, *Herc3*^*-/-*^ mice demonstrate differential expression of multiple genes involved in the modulation of microglial activation including *Edn2, Neat1, Meg3* and *Npy6r. Edn2* has been reported to be upregulated in injured photoreceptors, and to contribute to the activation of microglial cells^56^. *Neat1* is a long non-coding RNA that appears to promote the activation of the inflammasome in macrophages^57^ and to modulate microglial polarization towards the M1 phenotype^58^. *Meg3* seems to promote Nlrp3-mediated microglial inflammation by targeting miR-7a-5p^59^. *Npy6r* is a receptor for NPY (neuropeptide Y), which is known to have immunomodulatory, neuromodulatory and neuroprotective roles in the retina^60^. Finally, several complement genes were also upregulated in *Herc3*^*-/-*^ retinas. However, we did not find any severe dysregulation of inflammatory cytokines, consistent with the lack of overt inflammation in our model.

Herc3 is primarily located in the cytosol, in vesicle-like structures^61^ and endosomal/lysosomal compartments^62^. It may be involved in vesicular traffic and ubiquitin-dependent processes^63,64^. Herc3 is part of a family of Herc proteins that consist of two large (Herc1 and Herc2) and four small family members (Herc3-6)^40,50^. There is evidence suggesting that the large and small Herc molecules form two distinct families that are not derived from a common origin^40,50^. Interestingly, using single cell RNA sequencing we were able to show that most retinal cell types express Herc1, Herc2 and Herc3, but very little Herc4 and Herc6 (Herc5 is not expressed in mice). This means that Herc3 is the only small Herc molecule expressed to a significant extent in the retina, which may be one of the reasons for the lack of compensatory mechanisms for a Herc3 deficiency. As a side note, we did not enrich for microglia and cannot comment on their gene expression profile.

So far, no protein interactions have been identified for the RLD domain of Herc3^40^. Meanwhile, the HECT domain in Herc3 is functional and able to bind ubiquitin (and undergo ubiquitination)^61^. Within the last 2 years, this HECT domain of Herc3 has also been shown to ubiquitinate RPL23A^65^, EIF5A2^66^, and ERK2^67^ and target them for degradation. Herc3 may also have important biological functions that are independent of the RLD and HECT domains. Recent studies demonstrate that HERC3 may also be linked to inflammatory pathways by inducing the degradation of the RelA subunit of NF-κB and thus downregulating NF-κB signaling, independently of its E3 ligase activity^68^.

At this point, we can only speculate on the mechanism behind the retinal pathology caused by Herc3 deficiency. Photoreceptors normally have among the highest Herc3 expression levels in the retina, and photoreceptor cell body loss in the outer nuclear layer and accompanying loss of visual function are among the most prominent findings in Herc3 deficient mice. This suggests that the primary event may be affecting photoreceptors. One possibility is that ubiquitination of a key protein or set of proteins within photoreceptors (particularly rod photoreceptors) is specifically and uniquely carried out by Herc3, and thus absence of Herc3 leads to deficits in photoreceptor catabolic activities and cell loss. More work is needed in the future to further characterize which specific molecules are normally targeted by Herc3 in the retina. To this end, we plan to first study ERK2, EIF5A2, RPL23A, Ubiquilin-1/2 and NF-κB.

One limitation of our work was that we did not power our studies to fully establish whether there is an effect of sex on the retinal findings seen in Herc3 deficient mice. While both male and female *Herc3*^*-/-*^ mice develop retinal thinning and accumulation of fundus spots, we do see some evidence of a greater change in both of these variables in female mice. We plan to do further studies to try to determine if this is a reproducible finding and, if so, explore the mechanisms behind it. For these studies we will be collaborating with Dr. Katherine Wert, who is currently doing exciting work looking at the effects of sex on retinal dystrophies. Another limitation is that after inducing the Herc3 CRISPR mutation and breeding heterozygous mice twice, we proceeded to establish wild type and KO lines. However, this is unlikely to have had an impact in our findings for several reasons. First, maintaining a heterozygous x heterozygous breeding protocol is more important when generating new lines by crossing mutants with different backgrounds. In our case the mutation was induced in B6J mice and the line was maintained in that background. Second, Herc3 was targeted because in was identified in an unbiased ENU mutagenesis screen in which whole exome sequencing was done. Herc3 was found to be the only mutation in a pedigree that appeared to explain fundus spot accumulation and ONL thinning. After targeting Herc3 specifically using a completely different method (CRISPR) we again corroborated the exact same findings: fundus spot accumulation and ONL thinning. Thus, it is extremely unlikely that any other mutation is responsible for our findings. Finally, two separate CRISPR lines deriving from two separate founders had the exact same findings. Combining all of these pieces of information, we feel confident that the retinal degeneration and fundus spot accumulation we are observing are indeed secondary to the Herc3 mutation.

The work presented here confirms that using a fundus spot scale as a screening tool in a powerful forward genetics pipeline can identify important gene-retinal phenotype associations. It also specifically demonstrates that Herc3 is critical to retinal homeostasis. This is interesting because it shows that despite the large variety of E3 ubiquitin ligases expressed in the retina, they cannot substitute for Herc3 functionally. Further studies are needed to determine how the microglia are activated and if they have a role in the abnormalities seen. Using our forward genetics approach we have now identified several genes essential to retinal homeostasis. Based on these findings, we have so far developed new mouse models of retinal degeneration with different mechanisms of disease and clinico-pathological characteristics falling in very different parts of the spectra of fundus spot accumulation and outer retinal thinning (*Herc3*^*-/-*^ described here and Sfxn3^*-/-*^ published recently^47^). These models provide us with very interesting tools to explore not only the role of the specific genes in question, but also how microglial activation is associated with different types of retinal degeneration. To this end, in addition to model-specific interactome/proteomics experiments, we plan to apply gene expression and microglial depletion experiments to these mice. Also, applying models of diabetes and retinal injury to Herc3 deficient mice may shine some light on the potential impact of this specific E3 ubiquitin ligase in retinal diseases in which the ubiquitin proteasome system is thought to play an important role.

## Methods

All methods are reported in accordance with ARRIVE guidelines (https://arriveguidelines.org).

### Animals

All animal experiments were reviewed and approved by the UT Southwestern Institutional Animal Care and Use Committee (IACUC, protocol # 2015-G100937). Animals were handled in accordance with the ARVO Statement for the Use of Animals in Ophthalmic and Vision Research, and all international and NIH guidelines. Mice were euthanized by overdose of ketamine/xylazine (180 mg/kg, 24 mg/kg, respectively) followed by cervical dislocation. All mice were on the C57BL/6 J background and were free of the rd8 mutation. N-ethyl-N- nitrosourea (ENU) mutagenized mice, CRISPR/Cas9 generated *Herc3*^*-/-*^ mice and *Herc3*^+*/*+^ littermates were bred and maintained at the UT Southwestern Medical Center animal care facility (ARC) with 12 h light-on and 12 h light-off cycles. Mice were provided regular chow diet and water ad-libitum. Before all experiments, mice were anesthetized using a ketamine-xylazine cocktail (100 mg/kg-5 mg/kg) one at a time and eyes were dilated with a 1:1 mixture of tropicamide 1% solution and phenylephrine hydrochloride 2.5% solution (Alcon Laboratories, Inc., Fort Worth, TX, USA).

### ENU (N-ethyl-N- nitrosourea) mutagenesis, whole exome sequencing, and automated meiotic mapping

We used a robust and validated forward genetics protocol^47,48^ based on N-ethyl-N-nitrosourea (ENU)-mediated random mutagenesis (Fig. 1) in C57BL/6 J male mice, designated G0 (generation 0). G0 males were bred to wild-type C57BL/6 J female mice to produce G1 males, which are the founders of each pedigree. Whole exome sequencing was used to identify germline mutations in the G1 male founders, which were then bred to generate G2 and G3 mice (Fig. 1 of Wang et al.^48^). Each G3 mouse carries around 60 mutations. The zygosity of each mutation in G2 dams and in all G3 mice of each pedigree is determined before phenotypic screening by sequencing across pedigree-specific coding/splice site mutations using Ion Torrent AmpliSeq custom primer panels. In this work, the main phenotypic screen was fundus photography followed by scoring of yellow fundus spots using a special fundus spot scale (see below).

We then used Linkage Analyzer, an R-based analysis software, to detect statistically significant genotype–phenotype associations^48^. Linkage Analyzer performs automated computations of the P values for single-locus linkage for every mutation in the pedigree using recessive, semidominant (additive), and dominant transmission models. The magnitude of a quantitative or semi-quantitative phenotype is correlated with genotype at each mutation site for all mice in the pedigree. A Manhattan plot is then used to display the P values of genotype–phenotype associations for every mutation in the pedigree. In this plot − log_10_ P values (y-axis) are plotted vs. the chromosomal positions of the mutations (x-axis) that had been identified in the G1 founders of each pedigree. Mutations meeting the following two criteria were considered of interest: (1) their Manhattan plot peak is above a horizontal line representing a threshold of P = 0.05 with Bonferroni correction, and (2) the peak is at least 3 logs higher than the second highest peak in the pedigree. Such mutations were evaluated by a machine learning algorithm, Candidate Explorer, which predicts the likelihood that a mutation is truly causative of the phenotype in question^49^.

### Fundus photography and fundus spot grading

Fundus photographs of both eyes of each mouse in a given pedigree were obtained, as described before^4^, using a Micron IV retinal imaging microscope (Phoenix Micron, Inc. Bend, OR). To prevent corneal dehydration in the anesthetized mice, we first applied GenTeal liquid gel (Novartis, East Hanover, NJ, USA) to the ocular surface after pupil dilation. Following imaging, investigators were masked to the genotypic data of the mice and were tasked with grading the fundus photos using a modified version of a previously reported fundus spot scale^69^. Briefly, scoring was based on the presence of white/yellow fundus spots as follows: 0—no spots present, 1—one to ten spots present, 2—spots occupy approximately one quadrant of the fundus, 3—spots occupy approximately two-to-three quadrants of the fundus, 4—spots densely occupy all four quadrants of the fundus. The scores were then added for both eyes resulting in a range of 0 to 8 for the final score per mouse. These scores were then loaded into Mutagenetix and analyzed using Linkage Analyzer, which correlated these data with the genetic data and generated Manhattan plots.

### Generation of *Herc3*^*-/-*^ mouse lines

We generated two *Herc3* knock out (*Herc3*^*-/-*^) mouse lines using CRISPR-Cas9 technology as described before^47^. Briefly, super-ovulated female C57BL/6 J mice were mated overnight with C57BL/6 J male mice and fertilized eggs were collected the following day for injection with *Herc3* sgRNA and Cas9 mRNA. A total of 109 injected embryos were cultured in M16 medium (Sigma-Aldrich) at 37 °C in 5% CO_2_. To generate mutant mice, 86 two-cell stage embryos were transferred into the ampulla of the oviduct (10–20 embryos per oviduct) of pseudo-pregnant Hsd:ICR (CD-1) female mice (Harlan Laboratories). Seventeen pups were born, and 2 homozygous males were bred to C57BL/6 J females and further bred to produce two knockout lines containing the mutations shown in Supplementary Fig. S1. The first line, *Herc3*(T), had 1 bp insertion (T) in exon 14 of the *Herc3* gene that encoded a frame-shifted protein product beginning after amino acid 563 followed immediately by a premature stop codon. Similarly, the second line, *Herc3*(A), had 1 bp insertion (A) at the same location that also encoded a frame-shifted protein product beginning after amino acid 563 followed immediately by a premature stop codon. Both mutations are predicted to result in nonsense mediated decay of the transcripts and are considered null mutations. For the Herc3(A) line, one *Herc3*^*-/-*^ male was crossed with a B6J female to generate *Herc3*^+*/-*^ mice. For the Herc3(T) line one *Herc3*^*-/-*^ female was crossed with a B6J male to generate *Herc3*^+*/-*^ mice. For both lines, we bred *Herc3*^+/-^ males to *Herc3*^+*/-*^ females to generate a colony of *Herc3*^+*/*+^ and *Herc3*^*-/-*^ mice.

### Quantitative RT-PCR

To corroborate that the CRISPR-generated mice underwent nonsense-mediated decay of the *Herc3* transcript, we performed quantitative RT-PCR. Since the *Herc3*^*-/-*^ mice in this work are global knock out mice, and since *Herc3* is highly expressed in both brain and retina, we isolated RNA from the brains of mice used for other experiments in order to check *Herc3* transcript levels. Brain tissue (~ 15 mg, brain cortex) was harvested from *Herc3*^+*/*+^ (n = 3) and *Herc3*^*-/-*^ (n = 3) mice and flash frozen before mRNA extraction. The day of extraction, tissue was homogenized for 2 min on ice in 350 µL RNA extraction buffer (Aurum Total RNA Mini Kit, BioRad, Hercules, CA). Samples were then warmed to room temperature and spun (21,000xg) for 5 min, collecting the supernatant. All other extraction and purification steps were also performed at room temperature. RNA was purified by following the animal tissue protocol provided in the BioRad kit. The final elution volume was reduced to 20 µL to increase the final RNA concentration. RNA was then normalized to 275 ng and converted to cDNA in a 5 µL reaction (qScript SuperMix, QuantaBio, Beverly, MA). cDNA was diluted 20-fold to a final volume of 100 µL with MBG water prior to qPCR analysis. TaqMan probes (Life Technologies, Carlsbad, CA) spanning the Herc3 transcript (Mm07299202_m1, Herc3 exons 3–4; Mm00518565_m1, Herc3 exons 14–15; and Mm07299198_m1, Herc3 exons 20–21) were used to assess Herc3 expression, using beta-actin (Mm02619580_g1) as a housekeeping gene. qPCR was run on a QuantStudio 6 Real-Time qPCR system (Life Technologies) and analyzed by the accompanying software.

### Preparation of retina- and RPE-choroid-scleral flat mounts (“RPE flat mounts”), immunostaining and counting of subretinal microglia

Mice (4 *Herc3*^*-/-*^ and 4 control) were deeply anesthetized and both eyes were enucleated and fixed in 4% PFA at room temperature following a previously described protocol^4^. In brief, enucleated intact eyes were fixed in 4% PFA for 30 min, followed by an additional 30 min after removing the cornea. Then the retina and the RPE-choroid-sclera were separated and fixed for an additional 1 h. After washing 3 × 5 min in PBS, the RPE and retina flat mounts were double or triple stained at 4 ºC overnight (as detailed below) followed by incubation at RT for 2 h with the appropriate Alexa Fluor conjugated secondary antibodies. For microglia and infiltrating macrophage discrimination, we used a combination of anti-F4/80, anti-TMEM119, and anti-CCR2 antibodies^70^. For detection of microglial activation, we used a combination of anti-Iba1, anti-CD16. (See Supplementary Table S1 listing antibodies and dilutions).

For microglia counting, the Iba1-stained RPE flat mounts (4 *Herc3*^*-/-*^ and 5 *Herc3*^+*/*+^ mice) were observed under fluorescence microscopy at 20X magnification. Iba1 + cells were counted in selected fields at four quadrants (superior, inferior, nasal, and temporal) around the optic nerve head (ONH). Four 20X fields were selected in each of the central, paracentral, midperiphery, and periphery regions. The Iba1 + cell counts from the four fields from each of the four regions of four flat mounts (see diagram in Fig. 3a) were used for subretinal microglia analysis and comparison between the two genotypes. For morphological analysis both the RPE and retina flat mounts (4 *Herc3*^*-/-*^ and 5 *Herc3*^+*/*+^ mice) were imaged using a Leica TCS SP8 confocal laser scanning microscope equipped with a Leica Application Suite X, LAS X, software (Leica Microsystems Inc.). Images were taken either at low (25X) magnification or at high (63X) magnification using a sequential scanning method.

### Measurement of retinal thickness parameters on optical coherence tomography (OCT) images

After anesthesia and pupil dilation were achieved as described above, we obtained OCT images from both eyes using a Micron IV-OCT2 instrument (Phoenix-Micron, Inc) by placing a short (half-size) horizontal line scan two disc diameters superior to the optic disc. We then used ImageJ to measure the following parameters at the center of each image plus in two other locations (100 µm away from the center on each side): total retinal thickness (TRT), ganglion cell complex (GCC), outermost neural retina thickness (ONRT), and outer nuclear layer (ONL). Using the Straight-Line tool in ImageJ, TRT was measured from the top of Bruch’s membrane (BM) to the top of the internal limiting membrane (ILM). GCC was then measured from the bottom of the Inner Nuclear Layer (INL) to the top of the ILM. ONRT was measured from the top of BM to the top of external limiting membrane (ELM). We developed this ONRT parameter because we needed a parameter that did not include the ONL measurement and could help us detect photoreceptor health issues even in the absence of photoreceptor cell death. We performed an internal quality control test and found that the coefficient of variation (CV) for both intra-observer comparisons and inter-observer comparisons (1.3% and 1.0%, respectively) was very low using this ONRT parameter. Finally, we have published our work using this parameter (previously referred to as “ORT”)^47,69^. After sharpening the image to get better contrast, we measured the ONL from the top of the ELM to the bottom of the Outer Plexiform Layer (OPL). Finally, the three measurements for each parameter in an image were averaged and used for statistical analysis.

### Retinal thickness measurements on H&E-stained cross sections

Right eyes from deeply anesthetized *Herc3*^*-/-*^ and *Herc3*^+*/*+^ mice were collected and processed for histology as described before^3^. Hematoxylin and Eosin (H&E) staining of retinal sections were prepared for all samples (n = 6 per group). The entire length of H&E retinal sections was imaged at 20 × magnification on both sides of the Optic Nerve Head (ONH) using a Leica DM2000 Upright Compound microscope (Leica Microsystems, Wetzlar, Germany) equipped with an Optronics Microfire color CCD camera (Optronics, Goleta, CA, USA). The H&E images were opened in ImageJ and the ONL thickness was measured at 300 μm intervals starting from the ONH on either side. For each location, we took three measurements within a 20 μm distance from the 300 μm mark and averaged them. The number of layers of nuclei in the ONL was also counted at the same locations by using the duplicate tool in ImageJ. To this end, a rectangle was drawn consistently to one side of the 300 μm mark making sure to include three columns of cells per duplicate image. The number of cells in each of the three columns of nuclear layers was averaged at each 300 μm location. Thus, for each 300 μm location we reported two different parameters: # of nuclei per column of ONL, and ONL thickness in microns.

### Testing of visual function using the optomotor response

Optomotor testing was performed using the OptoMotry system (Cerebral Mechanics, Inc., Lethbridge, AB, Canada) in order to examine differences in visual threshold in *Herc3*^+*/*+^ (n = 4) vs. *Herc3*^*-/-*^ (n = 5) mice. Visual stimuli were displayed on four LCD screens placed around a central mouse stand according to the manufacturer’s protocol and previous publications^71,72^. Each mouse was tested individually while placed without restrain on the central stand. A visual stimulus consisting of a rotating vertical sine wave grating was presented to the mouse and the optomotor reflex was then recorded by manual tracking of head movements. To determine spatial frequency thresholds, an increasing staircase paradigm was utilized with 100% contrast. The highest spatial frequency (cycles/degree, c/d) generated in each direction was recorded as the single frequency threshold for each eye separately and the average of both eyes is reported for each mouse. All recordings were done by a masked investigator.

### Visual function analysis using electroretinography (ERG)

Retinal response to light in *Herc3*^*-/-*^ versus *Herc3*^+*/*+^ mice was recorded using a full-field ERG system (Celeris System, Diagnosys LLC, MA, USA). Procedures were conducted under dim red light. A group of 10-month-old mice (n = 12 per group) and a second group of 16–18-month-old mice (n = 4 *Herc*^+*/*+^ and n = 5 *Herc3*^*-/-*^) were dark adapted overnight for 12–16 h. Scotopic ERG recording was done on deeply anesthetized mice after pupil dilation. Each mouse was placed on the ERG console with the full-field stimulators/electrodes touching the eyes. The body temperature was kept at 37 °C on the console during the ERG procedure. Three standard parameters (a-wave, b-wave and oscillatory potentials) were recorded using ten sweeps. The ERG analysis of visual response was obtained in response to low (0.1 log cd.s.m^-2^) and moderate (1 log cd.s.m^-2^) flash intensities. The inter-stimulus interval was 0.7 s and 60 s for low and high flash intensities, respectively. The flash duration was 1 ms. A similar protocol, using 3 sweeps was used to measure c-waves in 16–18 m old mice (n = 12 *Herc*^+*/*+^ and n = 12 *Herc3*^*-/-*^). After 10 min of light adaptation at a setting of 3 log cd.s.m^–2^, the photopic ERG measurements were obtained at 3 and 10 log cd.s.m^-2^ flash intensities. Ten sweeps were recorded and averaged for each flash intensity. Data were analyzed using Diagnosys Espion Software (Diagnosys, Inc).

### Immunohistochemistry (IHC) of retinal cross sections

Eyes from 11- to 14-month-old *Herc3*^*-/-*^ and *Herc3*^+*/*+^ were prepared as described before^3^. Briefly, after anesthesia, right eyes were enucleated and frozen in isopentane that had been chilled by liquid nitrogen, then transferred to freeze substitution solution and stored at -80 degrees. After 48 h of freeze substitution, eyes were gradually thawed to room temperature for paraffin embedding^3^. Hematoxylin and eosin stain was performed to assess the morphology of the retina. Six-micrometer slices were deparaffinized in xylene, rehydrated in graded ethanol and stained with anti-Iba1 (Wako pure chemical, Cat. # 019-19741). Alexa Fluor-labeled secondary antibody to Iba1 was used to visualize subretinal microglia. DAPI was used for nuclear counterstaining. Immunofluorescence was seen and imaged using a Zeiss AxioObserver epifluorescence microscope (Hamamatsu Photonics, Middlesex, NJ, USA).

### Staining and quantitation of apoptotic cells (TUNEL) and cone-photoreceptors in retinal cross-sections

Retinal cross sections were prepared from freeze-substitution fixated eyes as described above (IHC of retinal cross sections). Five-micron sagittal oriented sections were sequentially cut by rotary paraffin microtomy according to established procedures and were used both for Terminal deoxynucleotidyltransferase-mediated UTP End Labeling (TUNEL, DeadEnd Fluorometric TUNEL System, Promega Cat # G3250) and for cone-photoreceptor staining. Retinal sections from four eyes from each group were subjected to TUNEL and counterstained with propidium iodide. These sections were imaged on confocal microscope (Leica DMI 6000 B, Leica Microsystems) at 25X magnification and the TUNEL positive cells were counted in 10–11 fields per group for quantitative analysis. To check for cone-photoreceptor loss we stained retinal sections with an antibody against cone arrestin (cat # AB15282, Millipore, an accepted marker for cone-photoreceptor cells). DAPI was used for nuclear counter staining. Three fields on either side of the optic disc were imaged (n = 4 eyes per group; n = 15–16 fields per group) and used for counting cone-arrestin + cells.

### Electron microscopy (EM) imaging and analysis

Left eyes from deeply anesthetized *Herc3*^*-/-*^ and *Herc3*^+*/*+^ mice were collected and processed for electron microscopy as previously described^47^. In brief, eyes were first fixed in 2% PFA and 2% glutaraldehyde in sodium cacodylate buffer followed by post-fixation in 1% osmium tetroxide. After trimming and dehydration in graded ethanol, the retina tissue was embedded in epoxy resin. The retina blocks were cut in slices of 70-nm-thickness and dyed with 2% aqueous uranyl acetate and lead citrate. The sections were then imaged using a JEOL 1200EX II transmission electron microscope (JEOL USA, Inc., Peabody, MA, USA). To compare the thickness of RPE layer between *Herc3*^*-/-*^ and *Herc3*^+*/*+^ mice, three measurements were taken at 200 µm intervals in the center of the section from BM to the apical end of the RPE. The thickness of basal infoldings was measured in a similar manner from BM to the tip of infoldings. We then counted the total as well as apical and basal number of mitochondria per EM field. We also analyzed the EM sections for two additional parameters: the number of melanosomes in RPE microvilli and the number of RPE phagosomes per EM field. The UTSW Electron Microscopy Core assisted with sectioning and imaging.

### Analyses of single-cell transcriptomes

The retinas from four 4-month-old C57BL/6 J mice were isolated and combined as we previously described (IOVS In Print and^47^). Single-cell suspensions were prepared using the Papain Dissociation System (Worthington Biochemical; catalog no. LK003150) according to the manufacturer’s instructions with minor modifications^47,73^. Dissociated single retinal cells were processed through the GemCode Single-Cell Platform using the GemCode Gel Bead, Chip, and Library Kits (10 × Genomics) according to the manufacturer’s protocol and with the help of the UT Southwestern Next-Generation Sequencing Core Facility. In brief, single cells were sorted into 0.4% BSA in Dulbecco’s PBS. Approximately 14,000 single retinal cells were added to a channel. The cells were then partitioned into Gel Beads in emulsion in the 10 × Genomics Chromium Controller, followed by cell lysis and barcoded oligo-deoxythymine priming and reverse transcription of polyadenylated RNA. Finally, amplified cDNAs were sheared for adapter and sample index attachment. Libraries were sequenced on an Illumina NextSeq 500. Cell Ranger 3.0.0 (10X Genomics) was used to process the raw sequencing data. BCL files were converted to FASTQ files and aligned to mouse (mm10) reference transcriptome. Transcript counts of each cell were quantified using UMI and valid cell barcode. The gene expression matrix from cell ranger was used as input to the Seurat R package (v3.0.0) for downstream analysis^74^. Cells with less than 200 genes per cell and high mitochondrial gene content were filtered out. The global-scaling normalization method “LogNormalize” was used for normalization. A subset of genes exhibiting high variation across the single cells was determined. The highly variable genes were calculated using the “FindVariableFeatures” module in Seurat. For the sample, a Shared Nearest Neighbor (SNN) Graph was constructed with the “FindNeighbors” module in Seurat by determining the k-nearest neighbors of each cell. The clusters were then identified by optimizing SNN modularity using the “FindClusters” module. This allowed for a sensitive detection of rare cell types. We obtained 23 clusters with a resolution of 0.5. Clusters were named on the basis of known gene markers specific to various cell types found in the retina^75^. Differential expression analysis of each cluster was performed in Seurat. Violin plots for *Herc* gene family were generated using Seurat.

### RNAscope in situ hybridization (ISH)

To determine the expression and localization of Herc3 in the retinal tissue we used a custom designed Herc3 Probe (Mm_Herc3 : cat#1300971, ACD Biotechne) for RNAscope ISH (Red chromogenic &amp; fluorescent kit, Advanced Cell Diagnostics, Hayward, CA USA) as described before (PMID: 32457148). A probe for SaCas9 was used as a negative control (Staphylococcus aureus Cas9, cat#501621, ACD Biotechne). After performing the RNAScope assay, images were taken on a Zeiss Axio Observer.D1 microscope at 20X magnification using an Axio Cam ICc1 camera (Carl Zeiss MicroImaging GmbH, Gottingen, Germany).

### Sample preparation for bulk RNA-seq

Both eyes from deeply anesthetized 8-month-old mice were collected and the neuroretina was isolated. Samples consisted of the combination of the retinas from both eyes (n = 3 *Herc3*^+*/*+^ and 4 *Herc3*^*-/-*^ samples). The samples were washed with PBS and kept in 200 µl RNAprotect Tissue Reagent (QIAGEN, #76104) at 4 °C until total RNA isolation. Neuroretina samples were homogenized using a Bel-Art Micro-Tube Homogenizer (Avantor, VWR, #76529-604) for 1 min. Homogenized lysate was then centrifuged for 3 min at 14,000 rpm and supernatant was collected for total RNA isolation using miRNeasy Tissue/Cells Advanced Micro Kit (QIAGEN, # 217684) according to the manufacturer’s protocol. On-column DNase digestion was carried out using RNAse-Free DNase Set (QIAGEN, #79254). Quantity and purity of isolated total RNA samples were determined by NanoDrop™ OneC Microvolume UV–Vis Spectrophotometer (Thermo Scientific™, #ND-ONEC-W). Sample quality control was performed by UTSW Medical Center Next Generation Sequencing Core using Agilent 2100 Bioanalyzer System to ensure RNA Integrity Number (RIN) ≥ 6.8. For bulk RNA sequencing, 1 µg total RNA/sample was submitted. Samples were sequenced on the Illumina NextSeq 500 with read configuration as 100 bp, single end reads.

### Bioinformatics analysis of bulk RNA sequencing data

The Fastq files were subjected to quality check using fastqc (version 0.11.5, http://www.bioinformatics.babraham.ac.uk/projects/fastqc) and fastq_screen (version 0.11.4, http://www.bioinformatics.babraham.ac.uk/projects/fastq_screen). FASTQ files were aligned to Mus musculus reference genomes (mm10, UCSC version) using STAR^76^ (v2.5.3a), a splice-aware aligner for RNA-seq data.

Read counts mapping to genomic feature for each sample was generated using featureCounts^77^ from the Rsubread package (v1.4.6). As the samples were sequenced in two different batches, we used ComBat-seq^78^ to account for the batch effects. Next, batch adjusted read count matrix was used to run the differential expression analysis using edgeR^79^. edgeR uses TMM normalization and estimates the dispersion of the negative binomial distribution from replicates in each group. Furthermore, edgeR applies the Benjamini–Hochberg method on the p-values to control for FDR. Statistical cutoffs of *p-value* < 0.01, log_2_CPM > 0 and − 0.58 > log_2_FC > 0.58 (fold change > 1.5) were used to identify differentially expressed genes with statistical significance. Pathway analysis was performed using IPA (https://www.qiagenbioinformatics.com/products/ingenuity-pathway-analysis/).The dot plots were generated using ggplot2 (https://ggplot2.tidyverse.org/reference/geom_dotplot.html). The heatmap was generated using complex heatmaps^80^. The volcano plot was generated using EnhancedVolcano (https://github.com/kevinblighe/EnhancedVolcano).

### Statistical analysis

Results are presented as mean ± standard error of mean (SEM) except for data in Fig. 1c, where standard deviation (SD) was used. Comparisons between two groups were done using two-tailed unpaired Student's t-test. Linear regression analysis was done to test trends in changes of fundus spots and OCT parameters with time. A P-value < 0.05 was considered statistically significant. For the analysis of Iba1 + cells in flat mounts we provide the P-value both including all values (black asterisks) and excluding outliers which were identified in the midperipheral fields (blue asterisks). For this purpose, we used the interquartile range (IQR) to determine outliers, which were defined as those outside of a range going from Q1–1.5*IQR to Q3 + 1.5*IQR, where Q1 and Q3 are the first and third quartiles, respectively.

### Ethics approval and consent to participate

All animal studies were reviewed and approved by the UT Southwestern Medical Center Institutional Animal Care and Use Committee.

### Supplementary Information

Supplementary Information.

Supplementary Table S3.

## Data Availability

All data supporting this work are provided within the Article and Supplementary Files, or available from the corresponding authors upon request.

## Code availability

No custom code or algorithms were generated in this study. Candidate explorer is publicly accessible at https://mutagenetix.utsouthwestern.edu/linksplorer/candidate.cfm.

## Material availability

Herc KO mice are available upon request according to the UT Southwestern Medical Center MTA guidelines.

## References

[1] Massengill MT (2018). Clinically relevant outcome measures for the I307N rhodopsin mouse: A model of inducible autosomal dominant retinitis pigmentosa. Investig. Ophthalmol. Vis. Sci..

[2] Altmann C, Schmidt MHH (2018). The role of microglia in diabetic retinopathy: Inflammation, microvasculature defects and neurodegeneration. Int. J. Mol. Sci..

[3] Aredo B (2015). A chimeric Cfh transgene leads to increased retinal oxidative stress, inflammation, and accumulation of activated subretinal microglia in mice. Investig. Ophthalmol. Vis. Sci..

[4] Aredo B (2015). Differences in the distribution, phenotype and gene expression of subretinal microglia/macrophages in C57BL/6N (Crb1 rd8/rd8) versus C57BL6/J (Crb1 wt/wt) mice. J. Neuroinflamm..

[5] Gupta N, Brown KE, Milam AH (2003). Activated microglia in human retinitis pigmentosa, late-onset retinal degeneration, and age-related macular degeneration. Exp. Eye Res..

[6] Indaram M (2015). 7-Ketocholesterol increases retinal microglial migration, activation, and angiogenicity: A potential pathogenic mechanism underlying age-related macular degeneration. Sci. Rep..

[7] Kezic JM, Chen X, Rakoczy EP, McMenamin PG (2013). The effects of age and Cx3cr1 deficiency on retinal microglia in the Ins2(Akita) diabetic mouse. Investig. Ophthalmol. Vis. Sci..

[8] Ma W, Zhao L, Fontainhas AM, Fariss RN, Wong WT (2009). Microglia in the mouse retina alter the structure and function of retinal pigmented epithelial cells: A potential cellular interaction relevant to AMD. PLoS One.

[9] Madeira MH, Rashid K, Ambrosio AF, Santiago AR, Langmann T (2018). Blockade of microglial adenosine A2A receptor impacts inflammatory mechanisms, reduces ARPE-19 cell dysfunction and prevents photoreceptor loss in vitro. Sci. Rep..

[10] Narayan DS, Ao J, Wood JPM, Casson RJ, Chidlow G (2019). Spatio-temporal characterization of S- and M/L-cone degeneration in the Rd1 mouse model of retinitis pigmentosa. BMC Neurosci..

[11] Nebel C, Aslanidis A, Rashid K, Langmann T (2017). Activated microglia trigger inflammasome activation and lysosomal destabilization in human RPE cells. Biochem. Biophys. Res. Commun..

[12] O'Koren EG (2019). Microglial function is distinct in different anatomical locations during retinal homeostasis and degeneration. Immunity.

[13] Rutar M (2011). Analysis of complement expression in light-induced retinal degeneration: Synthesis and deposition of C3 by microglia/macrophages is associated with focal photoreceptor degeneration. Investig. Ophthalmol. Vis. Sci..

[14] Silverman SM, Ma W, Wang X, Zhao L, Wong WT (2019). C3- and CR3-dependent microglial clearance protects photoreceptors in retinitis pigmentosa. J. Exp. Med..

[15] Wang NK (2013). Origin of fundus hyperautofluorescent spots and their role in retinal degeneration in a mouse model of Goldmann-Favre syndrome. Dis. Model. Mech..

[16] Zhao L (2015). Microglial phagocytosis of living photoreceptors contributes to inherited retinal degeneration. EMBO Mol. Med..

[17] Zhong X (2016). Fundus camera-delivered light-induced retinal degeneration in mice with the RPE65 Leu450Met variant is associated with oxidative stress and apoptosis. Investig. Ophthalmol. Vis. Sci..

[18] Piedade WP, Famulski JK (2021). E3 ubiquitin ligase-mediated regulation of vertebrate ocular development; new insights into the function of SIAH enzymes. Biochem. Soc. Trans..

[19] Kaarniranta K (2020). Mechanisms of mitochondrial dysfunction and their impact on age-related macular degeneration. Prog. Retin Eye Res..

[20] Ye Q (2022). The role of RAD6B and PEDF in retinal degeneration. Neuroscience.

[21] Lobanova ES (2018). Increased proteasomal activity supports photoreceptor survival in inherited retinal degeneration. Nat. Commun..

[22] Ando R (2014). Decreased proteasomal activity causes photoreceptor degeneration in mice. Investig. Ophthalmol. Vis. Sci..

[23] Lim D, Park CW, Ryu KY, Chung H (2019). Disruption of the polyubiquitin gene Ubb causes retinal degeneration in mice. Biochem. Biophys. Res. Commun..

[24] Kaarniranta K (2022). Autophagy in age-related macular degeneration. Autophagy.

[25] Mitter SK (2014). Dysregulated autophagy in the RPE is associated with increased susceptibility to oxidative stress and AMD. Autophagy.

[26] Blasiak J, Pawlowska E, Szczepanska J, Kaarniranta K (2019). Interplay between autophagy and the ubiquitin-proteasome system and its role in the pathogenesis of age-related macular degeneration. Int. J. Mol. Sci..

[27] Svikle Z (2022). Ubiquitin-proteasome system in diabetic retinopathy. PeerJ.

[28] Wang Y, Punzo C, Ash JD, Lobanova ES (2022). Tsc2 knockout counteracts ubiquitin-proteasome system insufficiency and delays photoreceptor loss in retinitis pigmentosa. Proc. Natl. Acad. Sci. U.S.A..

[29] Walden H, Podgorski MS, Schulman BA (2003). Insights into the ubiquitin transfer cascade from the structure of the activating enzyme for NEDD8. Nature.

[30] Glickman MH, Ciechanover A (2002). The ubiquitin-proteasome proteolytic pathway: Destruction for the sake of construction. Physiol. Rev..

[31] Ye Y, Rape M (2009). Building ubiquitin chains: E2 enzymes at work. Nat. Rev. Mol. Cell Biol..

[32] Deshaies RJ, Joazeiro CA (2009). RING domain E3 ubiquitin ligases. Annu. Rev. Biochem..

[33] Campello L, Esteve-Rudd J, Cuenca N, Martin-Nieto J (2013). The ubiquitin-proteasome system in retinal health and disease. Mol. Neurobiol..

[34] Shang F, Taylor A (2012). Roles for the ubiquitin-proteasome pathway in protein quality control and signaling in the retina: Implications in the pathogenesis of age-related macular degeneration. Mol. Aspects Med..

[35] Xu J, Zhao H, Wang T (2020). Suppression of retinal degeneration by two novel ERAD ubiquitin E3 ligases SORDD1/2 in Drosophila. PLoS Genet..

[36] Chen X, Kezic J, Bernard C, McMenamin PG (2013). Rd8 mutation in the Crb1 gene of CD11c-eYFP transgenic reporter mice results in abnormal numbers of CD11c-positive cells in the retina. J. Neuropathol. Exp. Neurol..

[37] Kim SY (2014). Deletion of aryl hydrocarbon receptor AHR in mice leads to subretinal accumulation of microglia and RPE atrophy. Investig. Ophthalmol. Vis. Sci..

[38] Luhmann UF (2009). The drusenlike phenotype in aging Ccl2-knockout mice is caused by an accelerated accumulation of swollen autofluorescent subretinal macrophages. Investig. Ophthalmol. Vis. Sci..

[39] Raoul W (2008). Lipid-bloated subretinal microglial cells are at the origin of drusen appearance in CX3CR1-deficient mice. Ophthalmic Res..

[40] Sanchez-Tena S, Cubillos-Rojas M, Schneider T, Rosa JL (2016). Functional and pathological relevance of HERC family proteins: A decade later. Cell. Mol. Life Sci..

[41] Hochrainer K (2005). The human HERC family of ubiquitin ligases: Novel members, genomic organization, expression profiling, and evolutionary aspects. Genomics.

[42] Charette JR (2017). A mutagenesis-derived Lrp5 mouse mutant with abnormal retinal vasculature and low bone mineral density. Mol. Vis..

[43] Krebs MP (2017). Mouse models of human ocular disease for translational research. PLoS One.

[44] Maddox DM (2011). An ENU-induced mutation in the Mertk gene (Mertknmf12) leads to a slow form of retinal degeneration. Investig. Ophthalmol. Vis. Sci..

[45] Pinto LH (2005). Generation, characterization, and molecular cloning of the Noerg-1 mutation of rhodopsin in the mouse. Vis. Neurosci..

[46] Weatherly SM (2022). Identification of Arhgef12 and Prkci as genetic modifiers of retinal dysplasia in the Crb1rd8 mouse model. PLoS Genet..

[47] Chen B (2020). Forward genetic analysis using OCT screening identifies Sfxn3 mutations leading to progressive outer retinal degeneration in mice. Proc. Natl. Acad. Sci. U.S.A..

[48] Wang T (2015). Real-time resolution of point mutations that cause phenovariance in mice. Proc. Natl. Acad. Sci. U.S.A..

[49] Xu D (2021). Thousands of induced germline mutations affecting immune cells identified by automated meiotic mapping coupled with machine learning. Proc. Natl. Acad. Sci. U.S.A..

[50] Marin I (2010). Animal HECT ubiquitin ligases: Evolution and functional implications. BMC Evol. Biol..

[51] Bryan JM (2018). Identifying core biological processes distinguishing human eye tissues with precise systems-level gene expression analyses and weighted correlation networks. Hum. Mol. Genet..

[52] Aghdam SY, Sheibani N (2013). The ubiquitin-proteasome system and microvascular complications of diabetes. J. Ophthalmic Vis. Res..

[53] Kaarniranta K (2023). Autophagy in age-related macular degeneration. Autophagy.

[54] Chen M, Xu H (2015). Parainflammation, chronic inflammation, and age-related macular degeneration. J. Leukoc. Biol..

[55] Zhao X (2015). Neuronal interleukin-4 as a modulator of microglial pathways and ischemic brain damage. J. Neurosci..

[56] Rattner A, Nathans J (2005). The genomic response to retinal disease and injury: Evidence for endothelin signaling from photoreceptors to glia. J. Neurosci..

[57] Zhang P, Cao L, Zhou R, Yang X, Wu M (2019). The lncRNA Neat1 promotes activation of inflammasomes in macrophages. Nat. Commun..

[58] Ni X (2020). Knockdown lncRNA NEAT1 regulates the activation of microglia and reduces AKT signaling and neuronal apoptosis after cerebral ischemic reperfusion. Sci. Rep..

[59] Meng J (2021). LncRNA-Meg3 promotes Nlrp3-mediated microglial inflammation by targeting miR-7a-5p. Int. Immunopharmacol..

[60] Santos-Carvalho A, Alvaro AR, Martins J, Ambrosio AF, Cavadas C (2014). Emerging novel roles of neuropeptide Y in the retina: From neuromodulation to neuroprotection. Prog. Neurobiol..

[61] Cruz C, Ventura F, Bartrons R, Rosa JL (2001). HERC3 binding to and regulation by ubiquitin. FEBS Lett..

[62] Hochrainer K, Kroismayr R, Baranyi U, Binder BR, Lipp J (2008). Highly homologous HERC proteins localize to endosomes and exhibit specific interactions with hPLIC and Nm23B. Cell. Mol. Life Sci..

[63] Rotin D, Kumar S (2009). Physiological functions of the HECT family of ubiquitin ligases. Nat. Rev. Mol. Cell Biol..

[64] Metzger MB, Hristova VA, Weissman AM (2012). HECT and RING finger families of E3 ubiquitin ligases at a glance. J. Cell Sci..

[65] Zhang Z (2022). HERC3 directly targets RPL23A for ubiquitination degradation and further regulates colorectal cancer proliferation and the cell cycle. Int. J. Biol. Sci..

[66] Zhang Z (2022). HERC3 regulates epithelial-mesenchymal transition by directly ubiquitination degradation EIF5A2 and inhibits metastasis of colorectal cancer. Cell Death Dis..

[67] Xu S (2022). A novel ERK2 degrader Z734 induces apoptosis of MCF-7 cells via the HERC3/p53 signaling pathway. Molecules.

[68] Hochrainer K (2015). The ubiquitin ligase HERC3 attenuates NF-kappaB-dependent transcription independently of its enzymatic activity by delivering the RelA subunit for degradation. Nucleic Acids Res..

[69] Zhu Y (2019). Mice with a combined deficiency of superoxide dismutase 1 (Sod1), DJ-1 (Park7), and Parkin (Prkn) develop spontaneous retinal degeneration with aging. Investig. Ophthalmol. Vis. Sci..

[70] Paolicelli RC (2022). Microglia states and nomenclature: A field at its crossroads. Neuron.

[71] Alam NM, Altimus CM, Douglas RM, Hattar S, Prusky GT (2015). Photoreceptor regulation of spatial visual behavior. Investig. Ophthalmol. Vis. Sci..

[72] Prusky GT, Alam NM, Beekman S, Douglas RM (2004). Rapid quantification of adult and developing mouse spatial vision using a virtual optomotor system. Investig. Ophthalmol. Vis. Sci..

[73] Choi HJ, Wang R, Jakobs TC (2018). Single-cell dissociation and characterization in the murine retina and optic nerve. Methods Mol. Biol..

[74] Stuart T (2019). Comprehensive integration of single-cell data. Cell.

[75] Macosko EZ (2015). Highly parallel genome-wide expression profiling of individual cells using nanoliter droplets. Cell.

[76] Dobin A (2013). STAR: Ultrafast universal RNA-seq aligner. Bioinformatics.

[77] Liao Y, Smyth GK, Shi W (2014). featureCounts: An efficient general purpose program for assigning sequence reads to genomic features. Bioinformatics.

[78] Zhang Y, Parmigiani G, Johnson WE (2020). ComBat-seq: Batch effect adjustment for RNA-seq count data. NAR Genom. Bioinform..

[79] Robinson MD, McCarthy DJ, Smyth GK (2010). edgeR: A bioconductor package for differential expression analysis of digital gene expression data. Bioinformatics.

[80] Gu Z, Eils R, Schlesner M (2016). Complex heatmaps reveal patterns and correlations in multidimensional genomic data. Bioinformatics.

