# Supplementary material for: E3 ubiquitin ligase Herc3 deficiency leads to accumulation of subretinal microglia and retinal neurodegeneration
Source: Sci Rep. 2024 Feb 6;14:3010. doi: 10.1038/s41598-024-53731-8 (PMC10847449; doi:10.1038/s41598-024-53731-8)
Supplement: Supplementary file 1 — Supplementary Information. [file 41598_2024_53731_MOESM1_ESM.pdf]

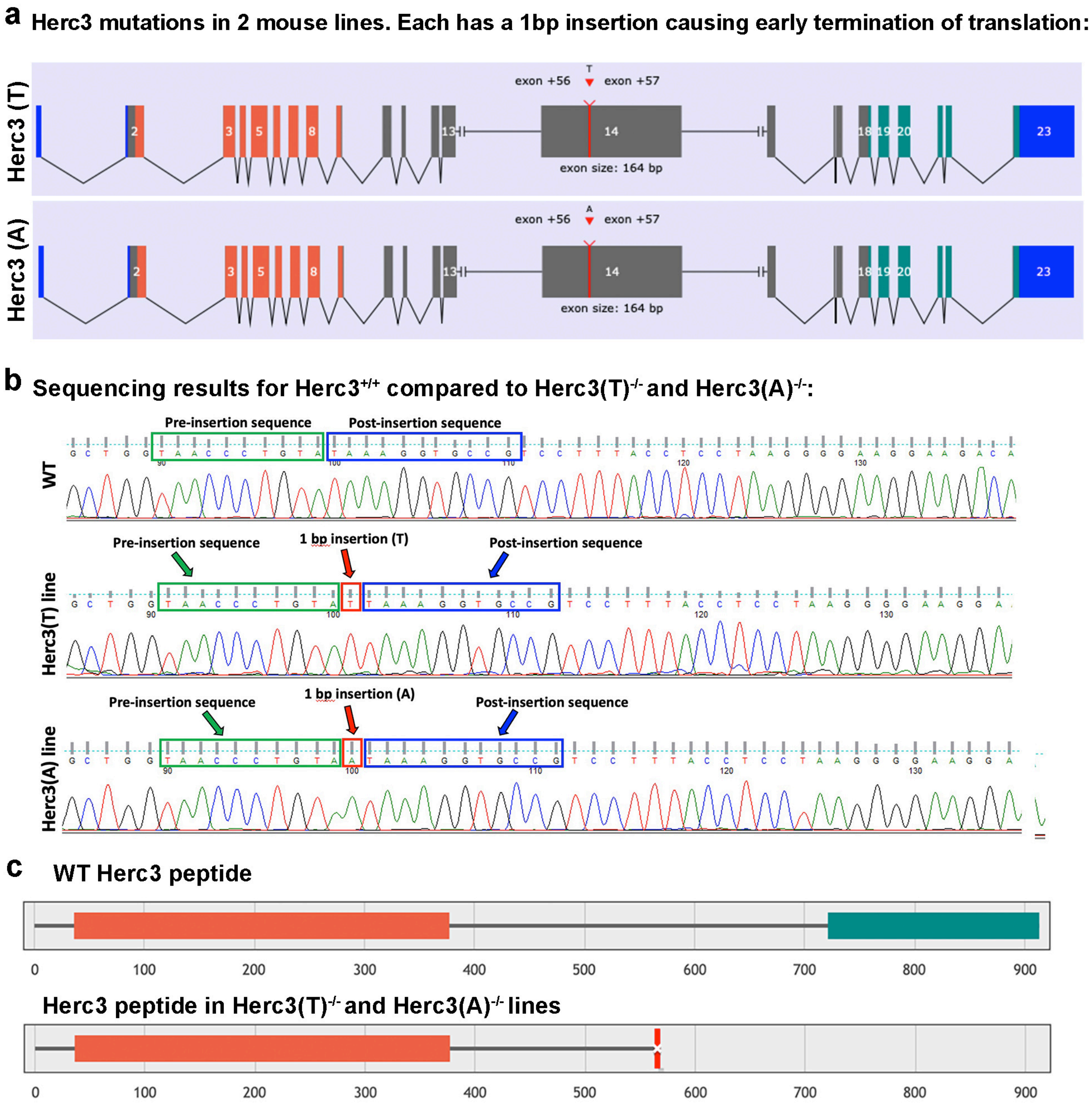

Supplementary Fig. S1: Genetic mutations, sequencing results and predicted peptides for two CRISPR-generated Herc3-/- mouse lines. a) The Herc3(T) mouse line has a 1 bp insertion (a thymine, T) after position 56 of exon 14, while the Herc3(A) line has a 1 bp insertion (adenine, A) at the same location. b) Examples of sequencing results for a WT mouse, a Herc3(T) KO and a Herc3(A) KO mouse are shown. c) In both mouse lines the mutations result in early termination of translation. In the schematic, the orange rectangle represents the RLD1 domain of Herc3, green represents the HECT domain, and red represents the stop codon. Please refer to Fig. 6a.

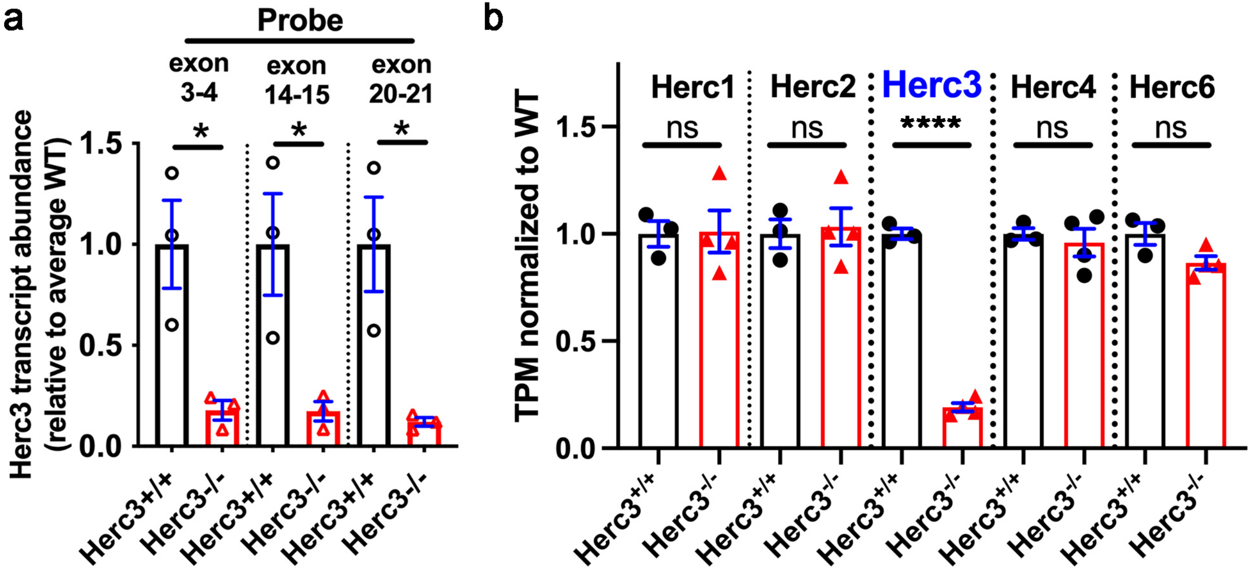

Supplementary Fig. S2: There is evidence of strong nonsense-mediated decay of Herc3 mRNA in Herc3-/- mice. a) Quantitative RT-PCR using three different TaqMan probes spanning the Herc3 transcript was done on cDNA from 3-5 m old Herc3+/+ (n=3) and Herc3-/- (n=3) mice. A strong and statistically significant decrease in transcript abundance in Herc3-/- samples was confirmed with all three probes. b) Bulk RNAseq analysis of retina samples from Herc3+/+ (n=3) and Herc3-/- (n=4) confirmed a marked reduction of Herc3 transcript in Herc3-/- retinas. No difference was detected in Herc1, Herc2, Herc4 or Herc6 transcripts. Herc5 was not detected. Two-tailed student's t-test: ns = no significant difference, *p<0.05, ****p<0.0001.

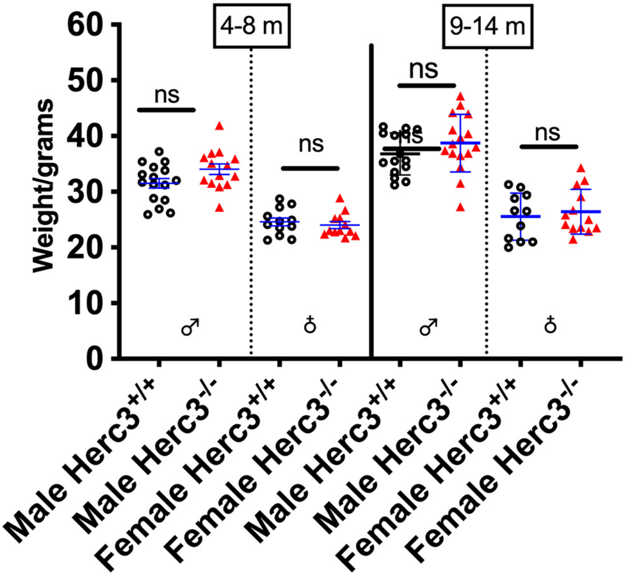

Supplementary Fig. S3: No weight abnormalities are seen in Herc3-/- mice compared to control. No statistically significant difference in the weight of Herc3 deficient mice compared to control mice was seen in either young (3-9 m) or older (9-15m) mice. Given the known differences in weight by sex, the comparisons were made by sex. Two-tailed student's t-test: ns = no significant difference. Number of mice used: Herc3+/+ (male 4-8 m, n=16; 9-14 m, n=14; female 4-8 m, n=12, 9-14 m, n=11); Herc3-/- (male 4-8 m, n=14, 9-14 m; female 4-8 m, n=12, 9-14 m, n=13).

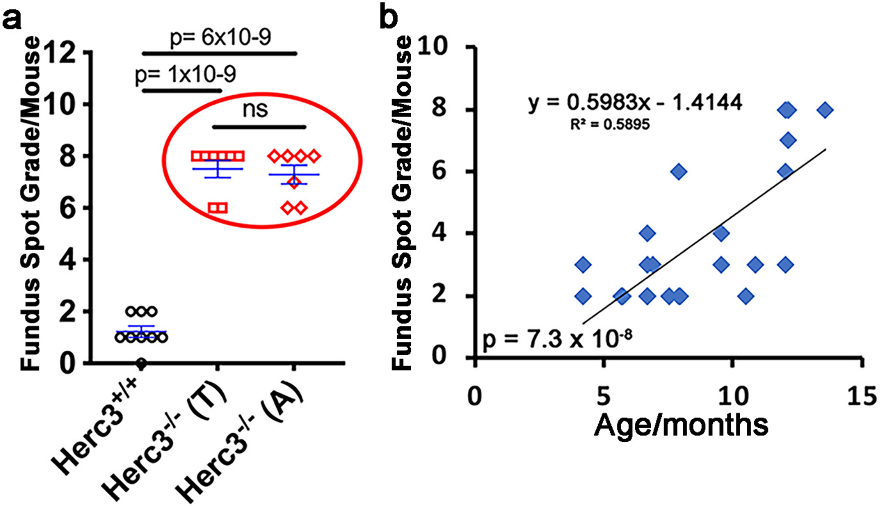

Supplementary Fig. S4: Fundus spot accumulation in Herc3-/- mice is similar in both transgenic lines. a) Both the Herc3(T) (n = 8) and the Herc3(A) (n = 7) lines show an increase in fundus spots accumulation compared to Herc3+/+ mice (n = 8). However, no difference between the two Herc3-/- lines was observed (red oval). Mice were 12–18-month-old, and each symbol represents an individual mouse. b) Linear regression analysis shows a statistically significant upward trend with age in fundus spots ($R2 = 0.5895$, $F = 47.4$, $p = 7.3 \times 10\text{-}8$). Two-tailed student's t-test: ns = no significant difference

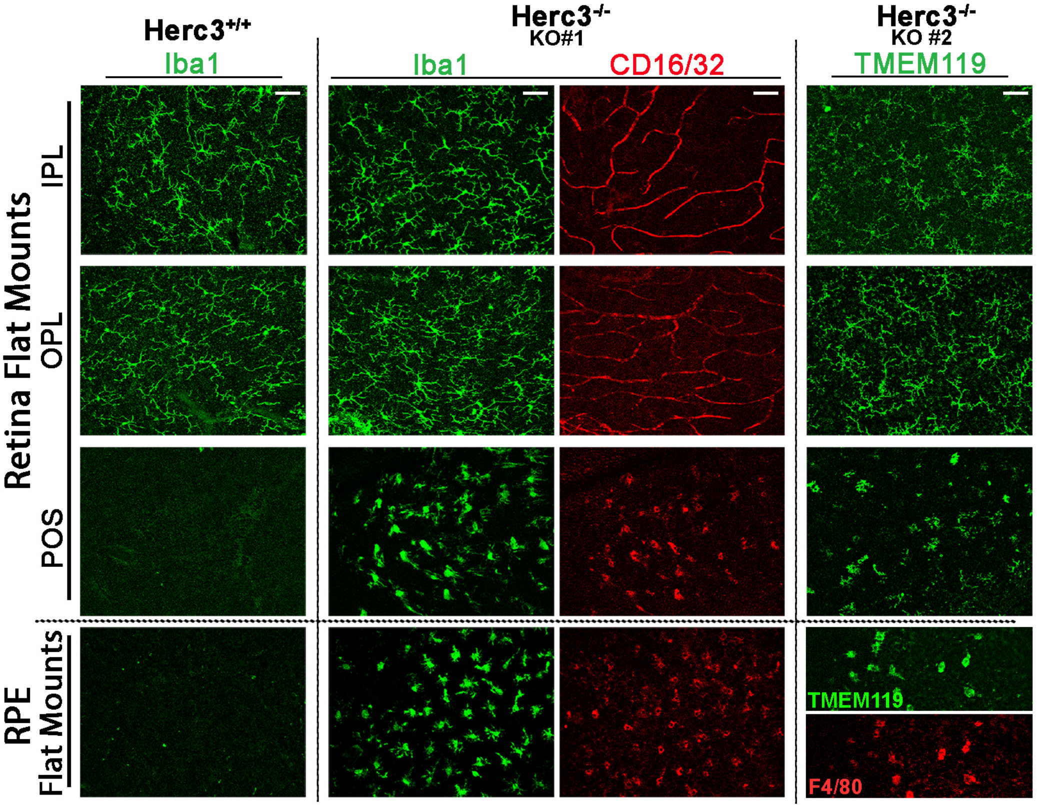

Supplementary Fig. S5: Herc3-/- mice accumulate activated microglia in the outer retina and subretinal space. Immunohistochemistry of retinal flat mounts (top 3 rows) and RPE flat mounts (bottom row) was performed on 15-17 m old Herc3+/+ (first column) and Herc3-/- (columns 2-3 are from one Herc3-/- mouse, and column 4 is a different Herc3-/- mouse) eyes. Herc3-/- retinas showed a similar distribution and appearance of microglia in the inner plexiform (IPL, first row) and outer plexiform layers (OPL, second row) compared to Herc3+/+. These cells were Iba1+, CD16-, and TMEM119+. They also had small cell bodies and long extensions. Interestingly, only Herc3-/- eyes showed accumulation of microglia in the photoreceptor outer segment layer (POS, third row) and the subretinal space (RPE flat mount, last row). The majority of cells in these two layers were were Iba1+, CD16+, and TMEM119+. These cells also show morphological signs of activation, with larger cell bodies and shorter extensions. The RPE flat mount on the last column (bottom panel) was co-stained with F4/80 (rather than Iba1) and TMEM. We did not find cells that were positive for F4/80 and negative for TMEM119, suggesting that most subretinal cells in our model were activated microglia rather than macrophages.

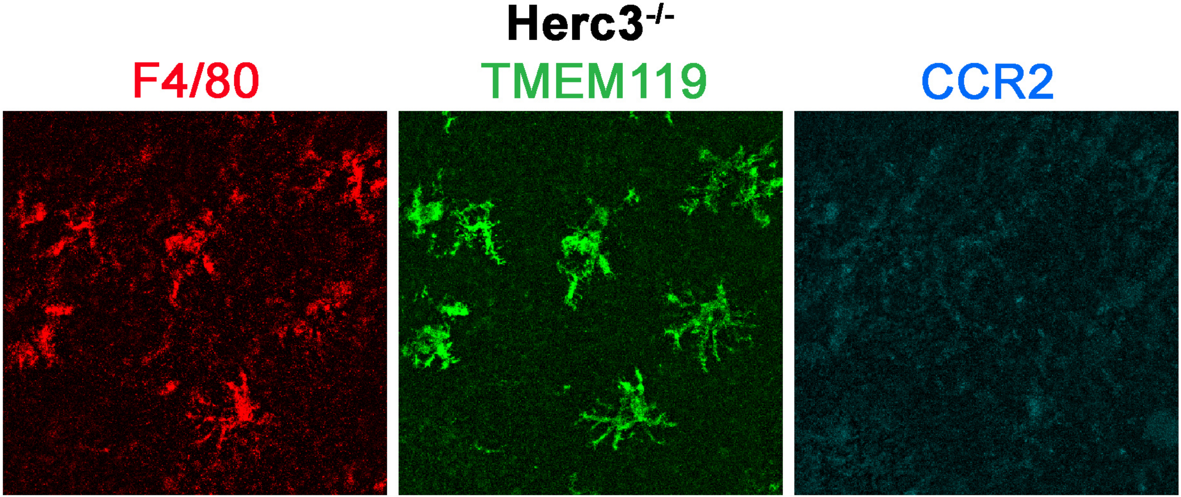

Supplementary Fig. S6: Subretinal cells stain with microglia but not infiltrating macrophage marker. Immunostaining of an RPE flat mount from a 16 m old mouse showing F4/80+ cells (pan-marker for microglia/macrophages) demonstrates that these cells also stain with a microglial-specific antibody (TMEM119), but not with a marker for infiltrating macrophages (CCR2).

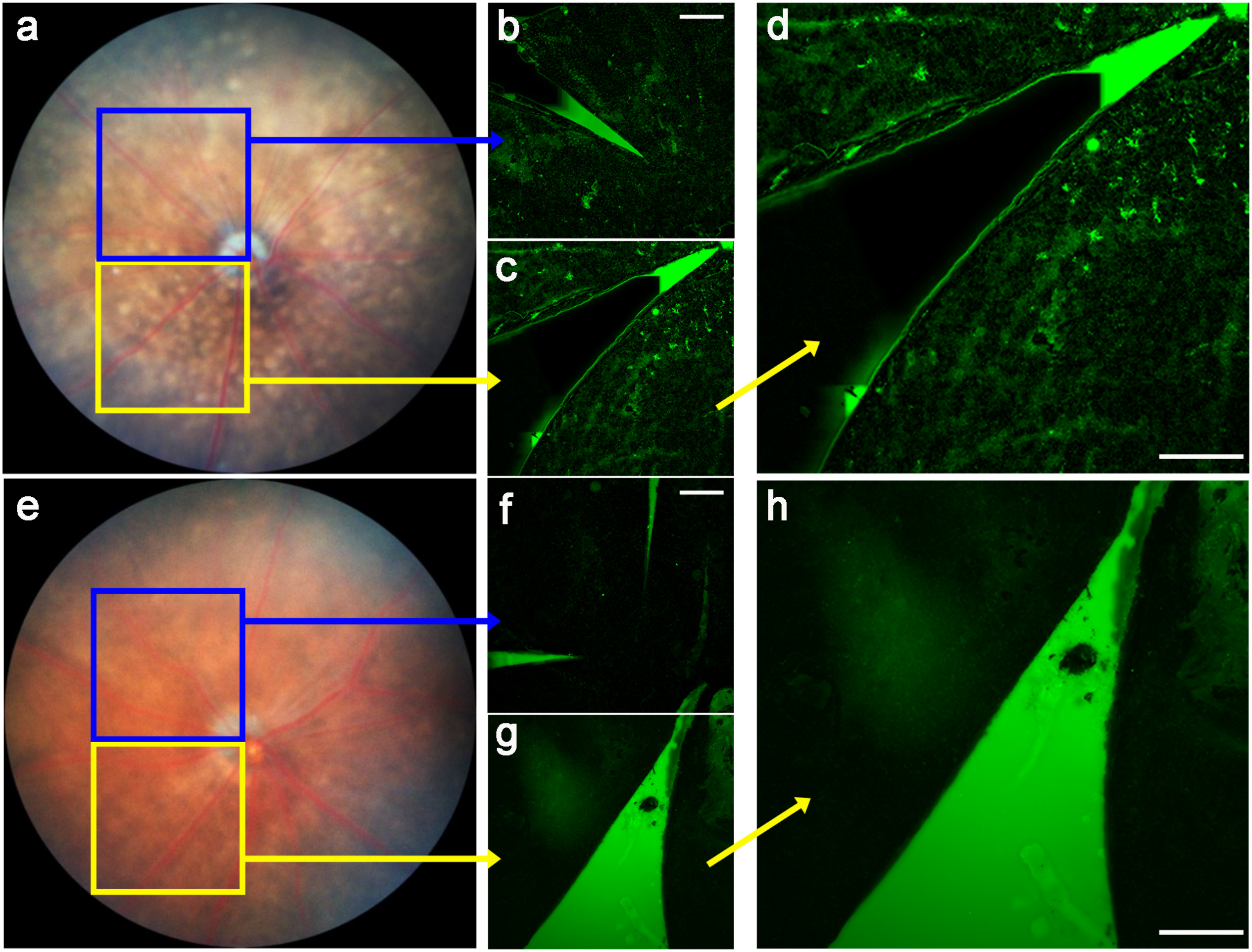

Supplementary Fig. S7: Representative fundus photographs of 8 m old Herc3-/- (a) and Herc3+/+ (e) mice are shown. The same eyes were collected for RPE/choroid/sclera flat mounts and stained with and anti-Iba1 antibody (b-d, f-h). While not common, we took advantage of this Herc3-/- mouse eye that showed sectoral accumulation of fundus spots. The flat mount areas in b and f correspond to the areas in blue squares in the left half of the fundus photographs in a and e, respectively, and show minimal fundus spots or Iba1+ cells. Meanwhile, the yellow square in the Herc3-/- eye (a) shows accumulation of fundus spots, while the corresponding area in the flat mount (c, and magnified view in d) shows accumulation of Iba1+ cells. No fundus spots or Iba1+ cells are seen in the corresponding Herc3+/+ fundus (e) and flat mount regions (g, h).

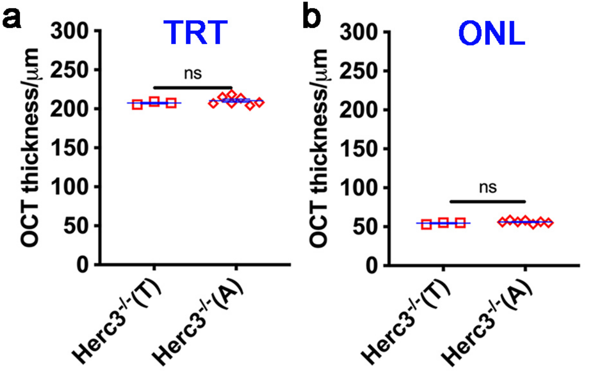

Supplementary Fig. S8: Retinal thickness on OCT in Herc3-/- mice is similar in both transgenic lines. When comparing the Herc3(T) mouse line and the Herc3(A) line, no difference was seen in either total retinal thickness (a) or ONL thickness (b). Mice were 9–15-month-old, and each symbol corresponds to an individual mouse. Two-tailed student's t-test: ns = no significant difference. Number of mice used: Herc3-/- (n=10).

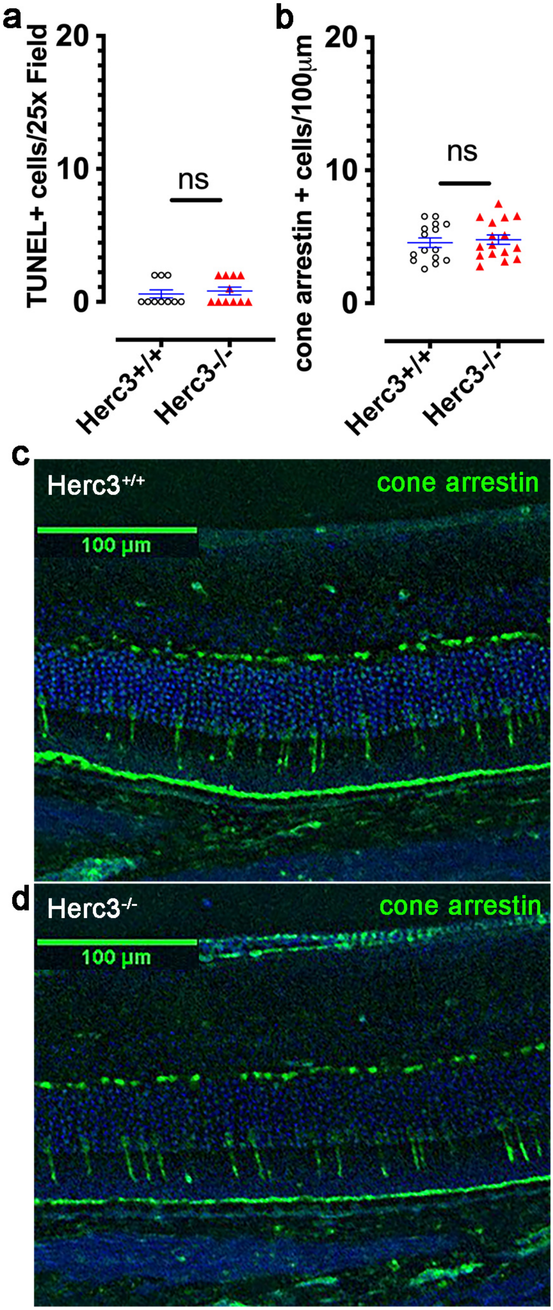

Supplementary Fig. S9: Apoptosis and cone density staining. a) A TUNEL assay was performed in both Herc3+/+ (n = 3 eyes) and Herc3-/- (n = 3 eyes) retinal sections. Three to four 25x-fields were imaged in each eye. The total number of TUNEL+ cells in each 25x-field image was graphed (n = 10 fields for Herc3+/+ and n = 11 fields for Herc3-/-). Very few TUNEL+ cells were seen, and there was no difference in the two mouse lines. b) Retinal sections from both Herc3+/+ (n = 3 eyes) and Herc3-/- (n = 3 eyes) mice were stained for cone arrestin and 4-6 fields (25x) per eye were imaged. Cone cells (cone arrestin+ cells) were counted in each image and the cell density (cells/100[illegible]m) for each image was calculated. The graph shows the cell densities for Herc3+/+ (n = 15) and Herc3-/- (n = 16) images. No difference was seen between the mouse lines. Representative cone arrestin images are shown for Herc3+/+ (c) and Herc3-/- (d) eyes.

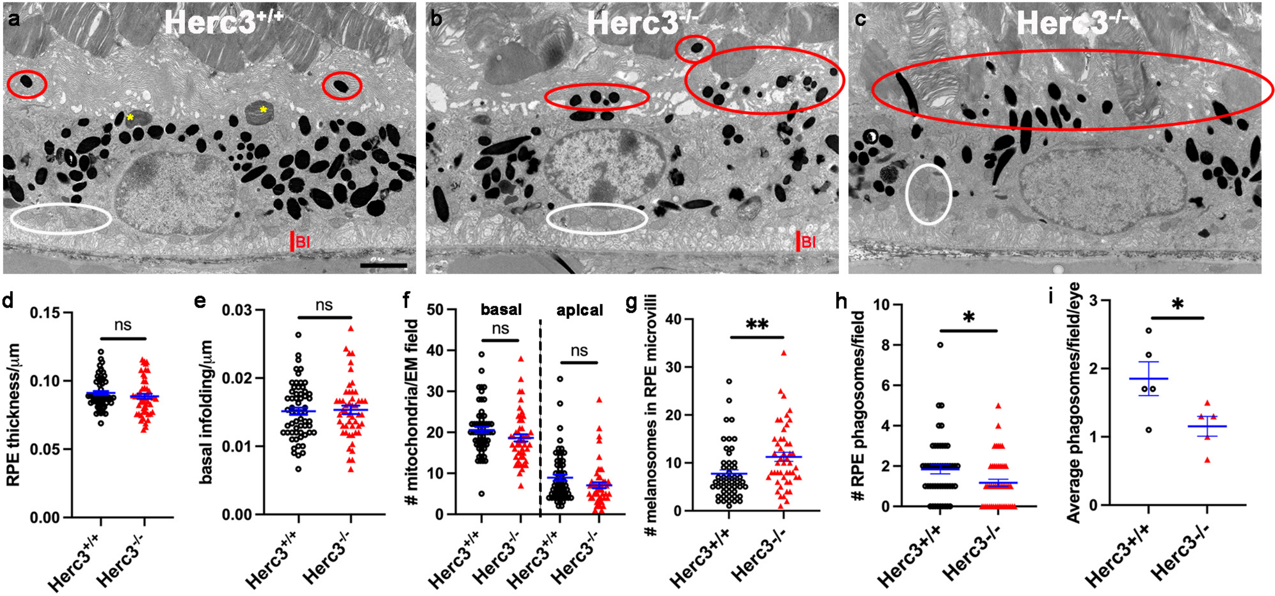

Supplementary Fig. S10. Electron Microscopy shows only mild RPE abnormalities in Herc3-/- mice. Representative EM images from a 10-m-old Herc3+/+ eye (a) and two age-matched Herc3-/- eyes (b,c). The yellow asterisks represent phagosomes. The melanosomes within the RPE microvilli are circled in red. The red lines correspond to basal infoldings (BI). The white ellipses highlight some mitochondria. RPE thickness (d) and basal infolding thickness (e) measurements on transmission electron microscopy (TEM) images show no significant differences between Herc3-/- and Herc3+/+ eyes. There are also no significant differences in the total number as well as apical or basal mitochondria in the RPE of Herc3-/- vs. Herc3+/+ eyes (f). On the other hand, we found a significant increase in the number of melanosomes dislocated into the RPE microvilli (g) and a significant decrease in the total number of RPE phagosomes per EM field (h) or the average number of phagosome per mouse in the Hrec3-/- mice compared to the Herc3+/+ mice. Data are shown as Means + SEM. EM fields (n = 50 per group). Number of mice (Herc3-/-, n = 5; Herc3+/+, n = 5). Two-tailed student's t-test: ns = no significance, $*p<0.05$, $**p<0.01$.

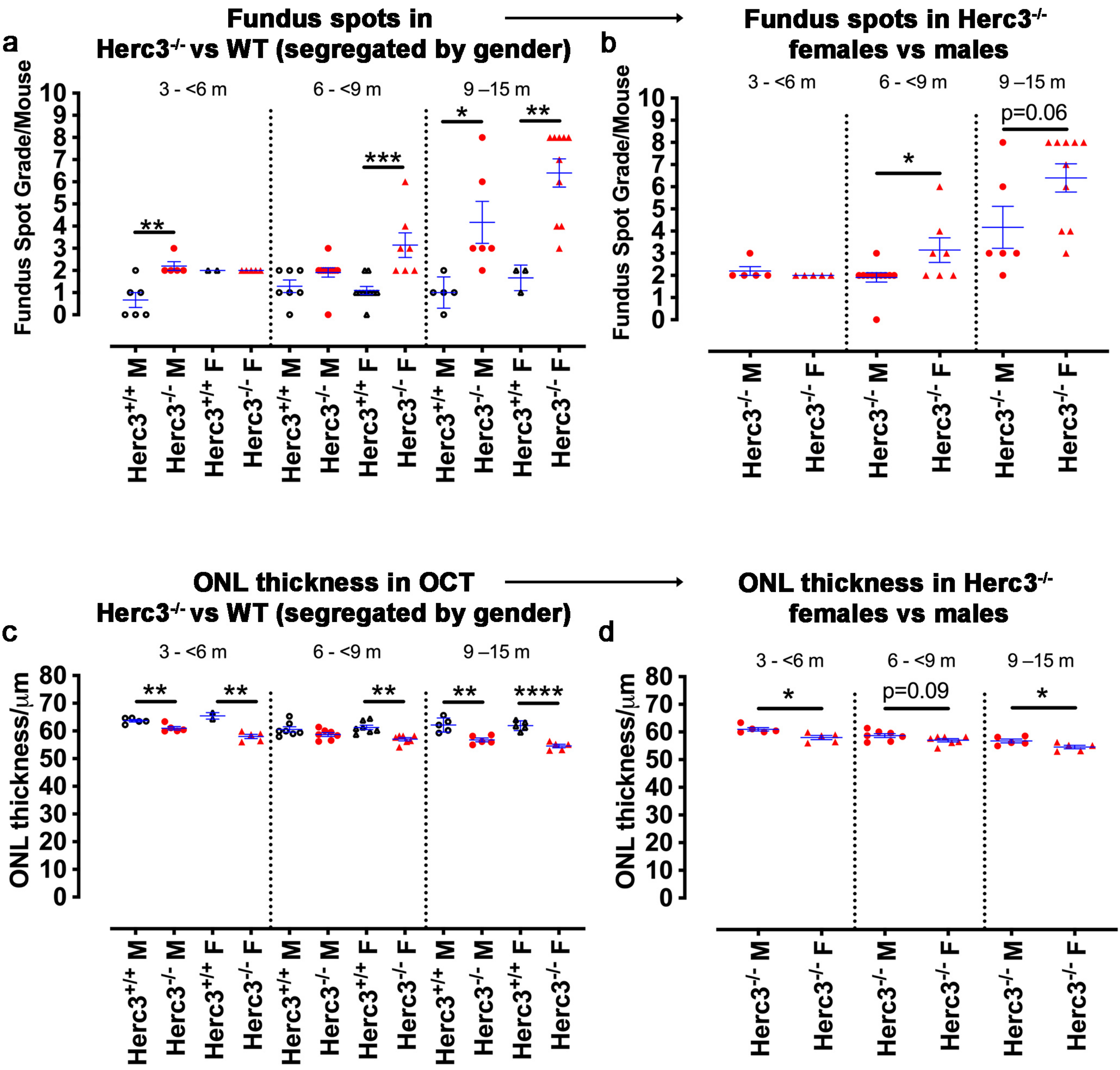

Supplementary Fig. S11. Potential impact of sex as a biological variable. An exploratory analysis of our fundus spot data (a,b) and our OCT data (c,d) after segregating mice by sex was performed. A statistically significant increase in fundus spots was seen in Herc3-/- compared to Herc3+/+ mice, independent of sex, for most comparisons (a). An increase in fundus spots in female Herc3-/- mice compared to male Herc3-/- mice was seen in some age groups (b). When looking at ONL thickness, Herc3-/- mice had significant thinning compared to Herc3+/+ independent of sex (c). Interestingly, we observed thinning of the ONL in female Herc3-/- mice compared to male Herc3-/- for two of the age groups (d). Two-tailed student's t-test: *p<0.05, **p<0.01, ***p<0.001, ****p<0.0001.

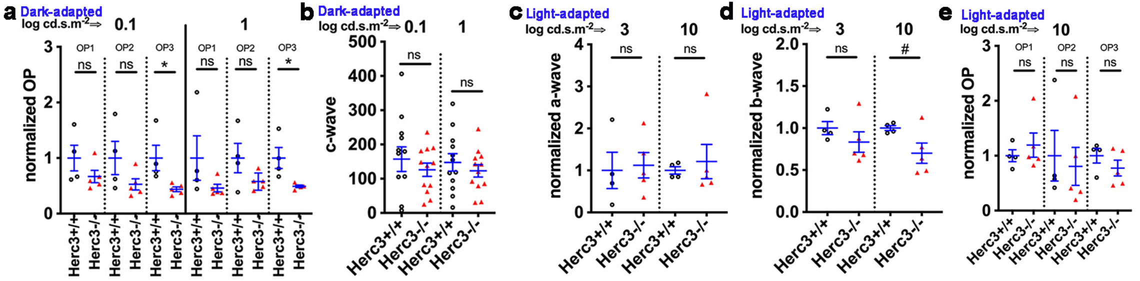

Supplementary Fig. S12. Minimal impact of Herc3 deficiency on c waves, oscillatory potentials and photopic ERG. Scotopic oscillatory potentials (a) and c waves (b) showed only minimal or no differences in Herc3-/- mice suggesting minimal or absent effects on the inner retina and RPE. Furthermore, the photopic a wave (c), b wave (d) and oscillatory potentials (e) also failed to show any significant changes in Herc3-/- mice. This would suggest that cones may not be as affected as rods, or that given the smaller number of cones, a difference may be harder to ascertain. Mice were 16-18 month-old. The experiments included 4 Herc3+/+ and 5 Herc3-/- mice, except for the c-wave data, which included 12 Herc3+/+ and 12 Herc3-/- mice. Data are shown as Means ± SEM. Two-tailed student's t-test: ns = no significant difference, # = p<0.1

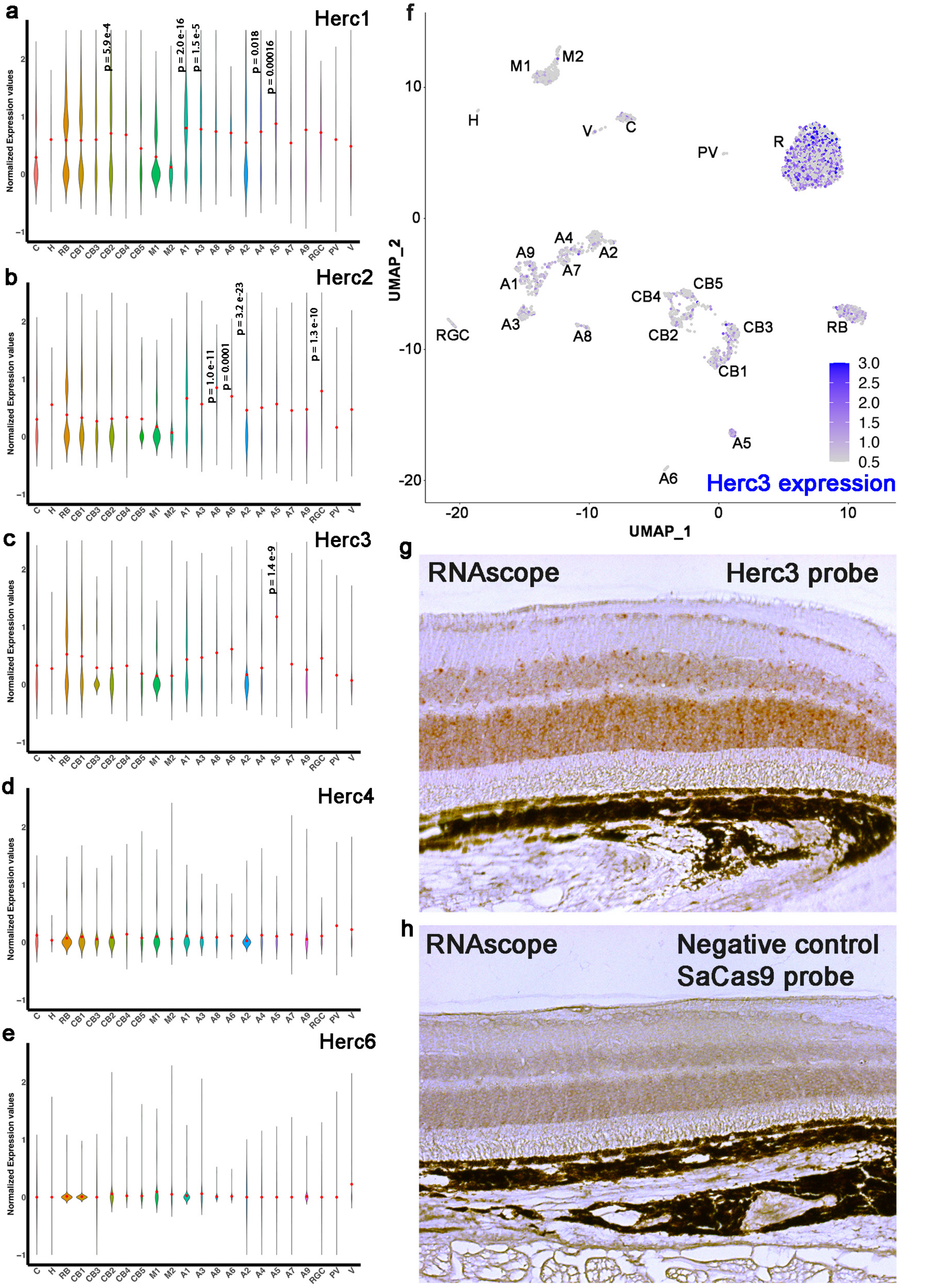

Supplementary Fig. S13: Single-cell RNA-seq data in C57BL/6J mice show ubiquitous expression of Herc3 in retinal cell types. Single-cell RNA sequencing data from 4-m-old B6J retinas. Average expression of Herc isoform transcripts in the major retinal cell types (excluding rods, which are shown in Fig. 6) are presented as violin plots. There is ubiquitous expression of the large Herc family members (Herc1 and Herc2) in many cell types (a and b). However, out of the three small Herc family members expressed in mice (Herc3, Herc4 and Herc6), Herc3 is the only one showing significant and ubiquitous expression in the retina (compare c vs. d and e). UMAP analysis showing the expression of Herc3 in the 23 clusters (f) illustrates the findings from the Herc3 violin plot (c). RNAscope for Herc3 in a wild type retinal section (g) confirms the ubiquitous expression of Herc3 in multiple retinal cell types and layers. As a negative control, a probe for SaCas9 (Staphylococcus aureus Cas9) was used (h). Abbreviations: C- cones, H- horizontal cells, RB- rod bipolar cells, CB- cone bipolar cells, M- Muller cells, A- amacrine cells, RGC- retinal ganglion cells, PV- perivascular cells, V- vascular endothelium.

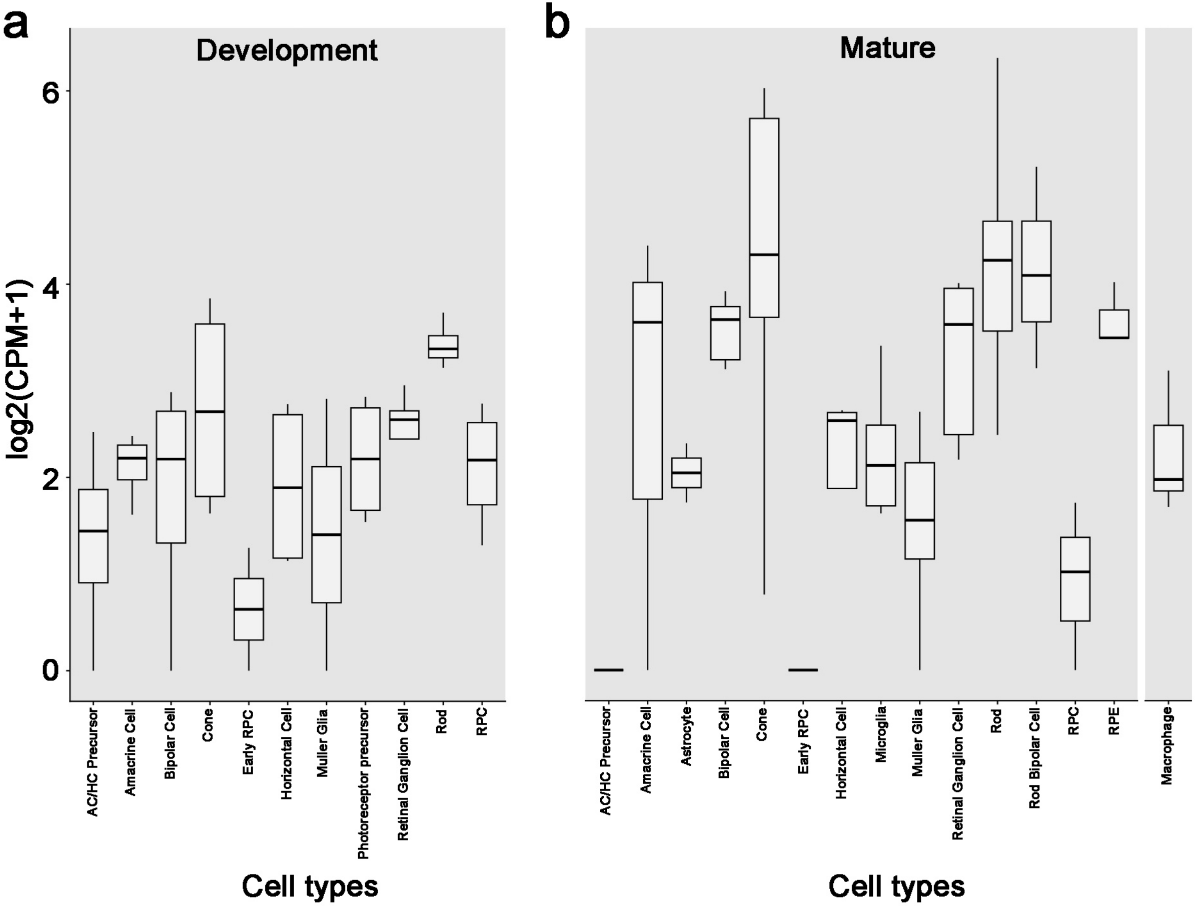

Supplementary Fig. S14: Expression of Herc3 at two developmental stages. The publicly available NEI database eyeIntegration (https://eyeintegration.nei.nih.gov) was used to search for the expression of Herc3 in the retina, both during development (a) and in adult mice (b). Box plots are shown for the different cell types.

Supplementary Table S1. Materials and Resources

Reagent Type	Name, description, dilution	Source, Reference	Cat. No
Antibody – Primary, Flat mount immunostaining, IHC	Anti-IBA1, Rabbit Polyclonal, 1:600	Wako Chemicals USA	019-19741
Antibody – Primary, Flat mount immunostaining	Anti-TMEM119, Rabbit Polyclonal1:200	Novus Biologicals, LLC	NBP3-13355
Antibody – Primary, Flat mount immunostaining	Anti-CCR2, Mouse Monoclonal, 1:100	Novus Biologicals, LLC	NBP2-35334
Antibody – Primary, Flat mount immunostaining	Anti-CD16/CD32, Rat Monoclonal, 1:25	BD Biosciences	553142
Antibody – Primary, Flat mount immunostaining	Anti-F4/80, Rat Monoclonal, 1:100	Santa Cruz Biotechnology	sc-52664
Antibody – Primary, Retinal cross-sections, IHC	Anti-Cone Arrestin, Rabbit Polyclonal, 1:250	EMD Millipore	AB15282
TUNEL Assay – Retinal cross-sections	DeadEnd Fluorometric TUNEL System	Promega	G3250
RNAscope Assay - Retinal cross-sections	RNAscope Probe- saCas9	Advanced Cell Diagnostics,Inc.	501621
RNAscope Assay - Retinal cross-sections	RNAscope Probe- Mm-Herc3-C1	Advanced Cell Diagnostics,Inc.	1300971
Antibody – Secondary, Flat mount immunostaining, IHC	Goat anti-Rabbit Alexa Fluor™ 633, 1:200	ThermoFisher Scientific	A21070
Antibody – Secondary, Flat mount immunostaining	Goat anti-Mouse IgG1 Alexa Fluor™ 488, 1:200	ThermoFisher Scientific	A21121
Antibody – Secondary, Flat mount immunostaining	Goat anti-rat IgG (H+L) Alexa Fluor™ 568, 1:200	ThermoFisher Scientific	A11077
Antibody – Secondary, Reytinal cross-sections, IHC	Donkey anti-Rabbit Alexa Fluor™ 488, 1:200	ThermoFisher Scientific	A21206

	# Cells	Herc1		Herc2		Herc3		Herc4		Herc6	
**Celltype**		**Mean**	**% exp.**	**Mean**	**% exp.**	**Mean**	**% exp.**	**Mean**	**% exp.**	**Mean**	**% exp.**
**A1**	167	0.798*	77†	0.663*	69†	0.433	49.1	0.108	16.8	0.026	4.2
**A2**	142	0.592†	40.1	0.514*	34.5	0.187	14.1	0.025	2.1	0.000	0.0
**A3**	89	0.779*	72†	0.564†	57†	0.467	53†	0.079	12.4	0.060	5.6
**A4**	71	0.764*	66†	0.502*	40.8	0.286	29.6	0.120	12.7	0.000	0.0
**A5**	52	0.875*	81†	0.565†	62†	1.21*	94†	0.102	15.4	0.000	0.0
**A6**	27	0.714†	85†	0.699*	89†	0.612†	78†	0.111	22.2	0.014	3.7
**A7**	28	0.540†	46.4	0.454	42.9	0.352	32.1	0.133	10.7	0.000	0.0
**A8**	41	0.738†	80†	0.849*	90†	0.549†	71†	0.086	14.6	0.011	2.4
**A9**	73	0.825*	53†	0.534†	37.0	0.256	21.9	0.051	4.1	0.013	1.4
**C**	97	0.290	27.8	0.299	29.9	0.327	27.8	0.119	12.4	0.000	0.0
**CB1**	191	0.585†	49.7	0.340	29.8	0.498	42.4	0.094	9.4	0.009	1.0
**CB2**	168	0.736*	53†	0.339	26.8	0.296	25.0	0.099	8.9	0.057	4.8
**CB3**	92	0.598†	50†	0.267	23.9	0.290	25.0	0.053	5.4	0.000	0.0
**CB4**	37	0.682†	62†	0.336	29.7	0.324	32.4	0.138	13.5	0.023	2.7
**CB5**	68	0.445	36.8	0.306	25.0	0.187	14.7	0.074	5.9	0.021	1.5
**H**	9	0.599†	89†	0.554†	89†	0.275	56†	0.033	11.1	0.000	0.0
**M1**	184	0.300	35.3	0.173	21.7	0.148	19.0	0.097	11.4	0.095	10.9
**M2**	61	0.159	11.5	0.111	6.6	0.189	9.8	0.059	3.3	0.050	3.3
**PV**	7	0.599†	57†	0.160	14.3	0.160	14.3	0.286	28.6	0.000	0.0
**R**	1516	0.256	13.9	0.191	11.1	0.77*	39.6	0.118	6.9	0.000	0.0
**RB**	237	0.589†	56†	0.378	39.7	0.523†	50†	0.070	8.0	0.015	1.7
**RGC**	39	0.721†	92†	0.790*	92†	0.454	72†	0.107	33.3	0.000	0.0
**V**	30	0.482	53†	0.470	53†	0.070	10.0	0.219	30.0	0.225	26.7

Supplementary Table S2. mRNA expression of Herc homologs in different retinal cell subtypes. Expression was mostly seen for *Herc1*, *Herc2* and *Herc3*. The asterisks (*) show cell subtypes that meet statistical significance for higher *Herc* expression compared to the average cell expression. The cross symbols (†) show cell types with an average expression greater than the arbitrary cut-off of 0.5 or for which > 50% cells express the given *Herc* molecule. Note that the only *Herc* homolog that is significantly expressed in rod photoreceptors is *Herc3* (black horizontal rectangle). Abbreviations: % exp. = % of cells expressing a given Herc, R- rods, C- cones, H- horizontal cells, RB- rod bipolar cells, CB- cone bipolar cells, M- Muller cells, A- amacrine cells, RGC- retinal ganglion cells, PV- perivascular cells, V- vascular endothelium. Mice were 4 m old.
